# Non-enzymatic *N*-acetylation of Lysine Residues by AcetylCoA Often Occurs via a Proximal *S*-acetylated Thiol Intermediate Sensitive to Glyoxalase II

**DOI:** 10.1016/j.celrep.2017.02.018

**Published:** 2017-02-28

**Authors:** Andrew M. James, Kurt Hoogewijs, Angela Logan, Andrew R. Hall, Shujing Ding, Ian M. Fearnley, Michael P. Murphy

**Affiliations:** 1 Mitochondrial Biology Unit, Medical Research Council, Cambridge CB2 0XY, UK; 2 Laboratory of Molecular Biology, Medical Research Council, Cambridge CB2 0QH, UK; 3 The Wellcome Trust Centre for Mitochondrial Research, Institute for Cell and Molecular Biosciences, The Medical School, Newcastle University, Newcastle upon Tyne NE2 4HH, UK

**Keywords:** AcetylCoA, lysine acetylation, glyoxalase

## Abstract

Acetyl coenzyme A (AcCoA), a key intermediate in mitochondrial metabolism, *N*-acetylates lysine residues, disrupting and, in some cases, regulating protein function. The mitochondrial lysine deacetylase Sirtuin 3 (Sirt3) reverses this modification with benefits reported in diabetes, obesity, and aging. We show that non-enzymatic lysine *N*-acetylation by AcCoA is greatly enhanced by initial acetylation of a cysteine residue, followed by *SN*-transfer of the acetyl moiety to a nearby lysine on mitochondrial proteins and synthetic peptides. The frequent occurrence of an *S*-acetyl intermediate before lysine *N*-acetylation suggests that proximity to a thioester is a key determinant of lysine susceptibility to acetylation. The thioesterase glyoxalase II (Glo2) can limit protein *S*-acetylation, thereby preventing subsequent lysine *N*-acetylation. This suggests that the hitherto obscure role of Glo2 in mitochondria is to act upstream of Sirt3 in minimizing protein *N*-acetylation, thus limiting protein dysfunction when AcCoA accumulates.

## Highlights

• AcCoA and acetylglutathione reversibly acetylate protein cysteine residues
• Non-enzymatic lysine acetylation proceeds via a proximal *S*-acetylated thiol intermediate
• Glyoxalase II and glutathione limit lysine *N*-acetylation and *N*-succinylation
• These findings have implications for *N*-acetylation of lysines in regulation and pathology

## Graphical Abstract

**Figure:**
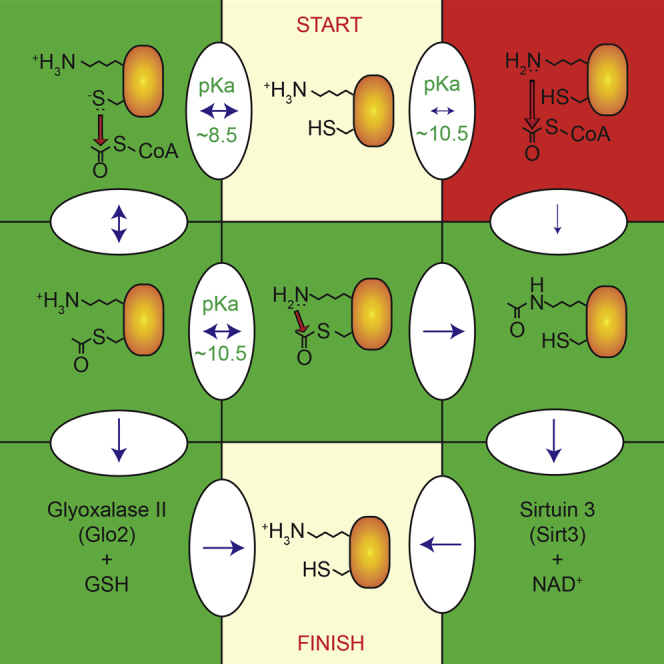

## Teaser

James et al. show that the non-enzymatic *N*-acetylation of lysine residues in mitochondrial proteins frequently occurs via a proximal *S*-acetylated thiol intermediate. Glutathione equilibrates with this intermediate, allowing the thioesterase glyoxalase II to limit protein lysine *N*-acetylation. These findings expand our understanding of how protein acetylation arises.

## Introduction

Acetyl coenzyme A (AcCoA) is central to mitochondrial metabolism, providing acetyl groups to the citric acid cycle from the oxidation of carbohydrate and fat (Pietrocola et al., 2015). One way that AcCoA affects mitochondria is through *N*-acetylation of the ε-amino of lysine residues on mitochondrial proteins. The significance of *N*-acetylation is implied by the existence of a mitochondrial deacetylase, sirtuin 3 (Sirt3), which uses NAD^+^ to remove acetyl groups from lysine residues, and by the observation that Sirt3 is important in the pathology of a range of degenerative diseases, including cancer, aging, and diabetes (McDonnell et al., 2015).

Although the enzymatic basis of the deacetylation of *N*-acetylated lysine residues by Sirt3 is established, the mechanism by which mitochondrial AcCoA acetylates lysine residues is less certain. Although a mitochondrial *N*-acetyltransferase (Gcn5L1) has been proposed (Scott et al., 2012), more interesting in the context of degenerative disease is the observation that mitochondrial protein is non-enzymatically *N*-acetylated on lysine residues by AcCoA (Wagner and Payne, 2013, Wagner and Hirschey, 2014, Weinert et al., 2015, Davies et al., 2016). The mitochondrial concentration of coenzyme A (CoA) in vivo (∼1 mM) is usually higher than AcCoA (∼100–500 μM) (Garland et al., 1965), but this shifts in the presence of fatty acids with AcCoA rising (∼1 mM) and CoA falling (∼100–300 μM). Consequently, both the mitochondrial concentration of AcCoA and the AcCoA/CoA ratio alter markedly upon changes to nutrition or exercise. Whether protein lysine acetylation is a damaging consequence of relying on AcCoA for metabolism or a regulatory pathway to respond to changes in AcCoA, the AcCoA/CoA ratio or NAD^+^ is unclear. Even so, Sirt3 plays a major pathophysiological role in reversing the damaging lysine *N*-acetylation of mitochondrial proteins that arises from exposure to excess AcCoA.

The mechanism proposed for non-enzymatic lysine acetylation was a nucleophilic attack by the side-chain amine on the acetyl carbonyl (Wagner and Payne, 2013). This direct reaction will be slow in the mitochondrial matrix (pH ∼7.8), because the pK_a_ of a free lysine is ∼10.5. Although this rate can be enhanced by stabilization of the amine, protein cysteine residues are on average better nucleophiles (pK_a_ ∼8.5) (Thapa et al., 2014). Because cysteine-containing peptides can be directly *S*-acetylated by AcCoA in vitro (Bizzozero et al., 2001), we postulated that *S*-acetylation of protein cysteine thiols would be far higher than *N*-acetylation of lysine amines. Here we show that *S*-acetylation is a frequent modification and that non-enzymatic *N*-acetylation of lysine residues by AcCoA occurs predominantly via a proximal *S*-acetylated thiol intermediate. We also show that glutathione (GSH) and glyoxalase II (Glo2), also known as hydroxyacyl glutathione hydrolase (HAGH), together can limit *S*-acetylation and consequently *N*-acetylation.

## Results

### Mitochondrial Proteins Are Acetylated by AcCoA

**Figure 1:**
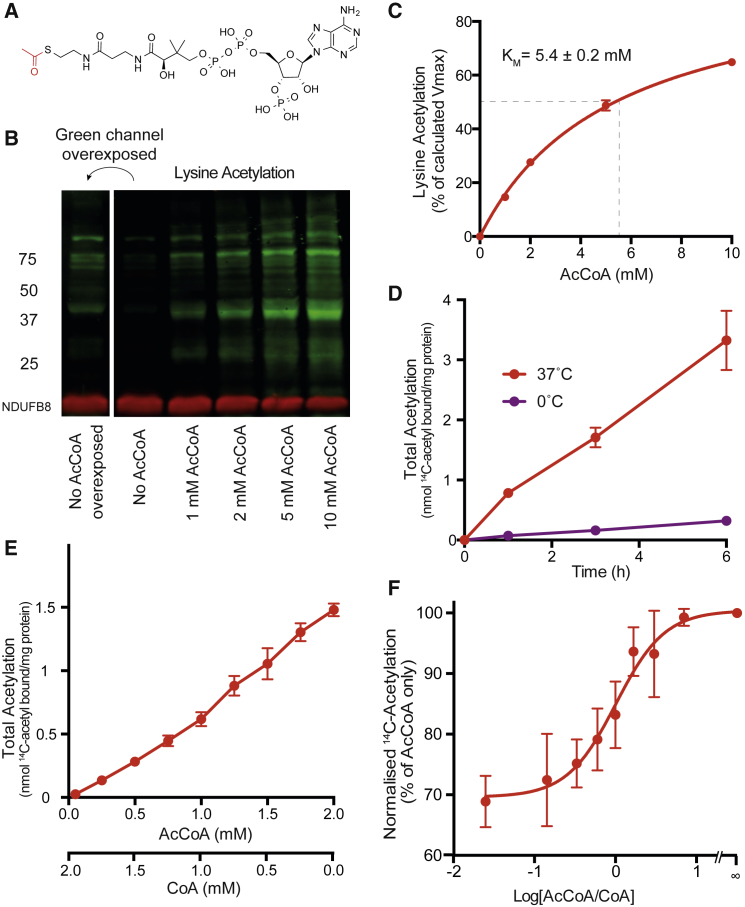
AcCoA Non-enzymatically Acetylates Mitochondrial Protein (A) Structure of AcCoA. (B and C) Lysine acetylation by AcCoA is concentration dependent, has a high apparent K_M_, and resembles in vivo acetylation. (B) Mitochondrial membranes were incubated with AcCoA at 37°C for 6 hr, followed by reducing SDS-PAGE. The green channel without AcCoA was overexposed for comparison. (C) After SDS-PAGE, acetyllysine (green) and NDUFB8 (red) were visualized (n = 2 ± range). (D) Total acetylation is temperature sensitive. Mitochondrial membranes were incubated with 2 mM ^14^C-AcCoA on ice or at 37°C. (E) Total acetylation is concentration dependent. Mitochondrial membranes were incubated with 50 μM ^14^C-AcCoA and 0–2 mM AcCoA at 37°C. The reaction contained sufficient CoA to keep AcCoA+CoA at 2 mM. Bound acetyl was calculated using the combined specific activity of ^14^C-AcCoA and AcCoA. (F) Acetylation is influenced by CoA. Incubations as for (E) but recalculated as ^14^C-acetyl bound (dpm) irrespective of specific activity. This was normalized to 2 mM AcCoA (∞). All data are the mean ± SEM of at least three experiments unless otherwise stated. See also Figure S1.

It has been shown using an anti-acetyllysine antibody that mitochondrial proteins can be non-enzymatically *N*-acetylated on lysine residues by AcCoA (Wagner and Payne, 2013). To explore this further, we incubated bovine heart mitochondrial membrane fragments that lack metabolites or matrix proteins with AcCoA. Lysine acetylation was concentration dependent (Figure 1B) in the physiological range for AcCoA with an apparent K_M_ of 5.4 mM (Figure 1C). The anti-acetyllysine antibody recognizes acetyllysine modifications (Figures S1A and S1B), and the pattern of lysine acetylation observed in this in vitro system resembles that of in vivo lysine acetylation (Figure 1B).

The mechanism proposed for lysine *N*-acetylation is via nucleophilic attack on AcCoA by the side-chain amine of a lysine residue (Wagner and Payne, 2013). However, the pK_a_ of a lysine amine is ∼10.5, making the more nucleophilic −NH_2_ form a minor species in vivo without stabilization. In contrast, cysteine residues are better nucleophiles at physiological pH, with significant amounts of the thiolate available for reaction (pK_a_ ∼8.5). Consequently, a major proportion of protein acetylation may be invisible to the anti-acetyllysine antibodies commonly used. To investigate total acetylation of mitochondrial protein by AcCoA, we incubated mitochondrial membranes with ^14^C-AcCoA. As expected, membrane-associated ^14^C increased with time and temperature (Figure 1D), consistent with a reaction between AcCoA and protein. The in vivo concentration of AcCoA plus CoA is likely to be stable ∼1–2 mM (Garland et al., 1965). To reflect this, we incubated mitochondrial membranes with 50 μM ^14^C-AcCoA supplemented with varying ratios of AcCoA and CoA to a total concentration of 2 mM AcCoA+CoA. The rate of protein acetylation was a function of the AcCoA concentration (Figure 1E) and the AcCoA/CoA ratio (Figure 1F) in the physiological range for AcCoA.

Thus, acetylation of mitochondrial proteins occurs in vitro in a time-, temperature-, and concentration-dependent manner without matrix proteins, other water-soluble metabolites, or cofactors. It has a high apparent K_M_, and the pattern of proteins modified resembles in vivo acetylation.

### Mitochondrial Proteins Are *S*-acetylated by AcCoA

**Figure 2:**
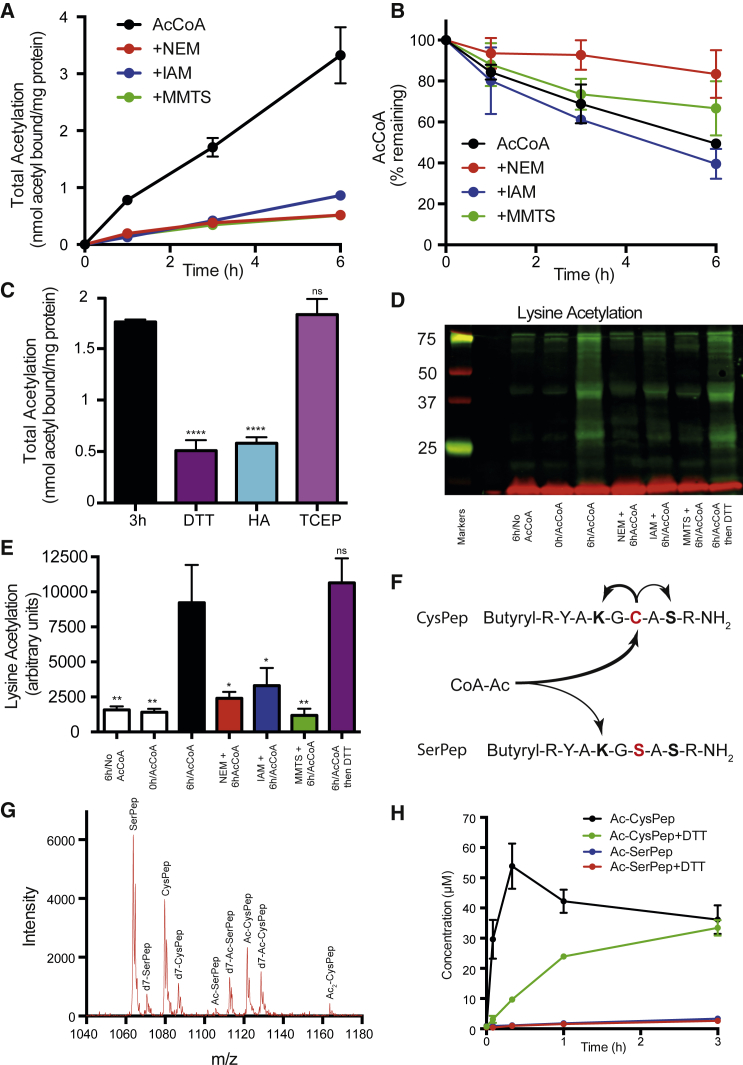
AcCoA Acetylates Lysine Residues via a Proximal Acetylcysteine Intermediate (A) Total acetylation is sensitive to thiol-blocking reagents. Mitochondrial membranes were incubated with 2 mM ^14^C-AcCoA and 25 mM NEM, IAM, or MMTS at 37°C for up to 6 hr. (B) Alkylating reagents do not break down AcCoA. Mitochondrial membranes were incubated with 2 mM AcCoA and 25 mM NEM, IAM, or MMTS at 37°C for up to 6 hr. AcCoA was measured by liquid chromatography-tandem mass spectrometry (LC-MS/MS) (n = 2 ± range). (C) Total acetylation is sensitive to thioester-cleaving reagents. Mitochondrial membranes were incubated with 2 mM ^14^C-AcCoA at 37°C for 3 hr. Afterward, 20 mM DTT, HA, or TCEP was added for 30 min at 37°C. (D and E) *N*-acetylation is sensitive to thiol-blocking reagents. (D) Mitochondrial membranes were incubated with 2 mM AcCoA and 25 mM NEM, IAM, or MMTS at 37°C for 6 hr. For others, 20 mM DTT was added for 30 min after the reaction. After reducing SDS-PAGE, acetyllysine (green) and NDUFB8 (red) were visualized. (E) The extent of lysine acetylation was quantified (n = 3 ± SD). (F–H) Proximal thiol facilitates lysine acetylation as is shown in (F). A cysteine-containing peptide (CysPep; 200 μM) and a serine-containing peptide (SerPep; 200 μM) were coincubated with 200 μM AcCoA. To differentiate *S*-acetylcysteine from *N*-acetyllysine and *O*-acetylserine, 20 mM DTT was added for 30 min after the reaction. A mix of deuterated (d7) standards was then added to quantify acetylation (Ac-SerPep and Ac-CysPep) by MALDI-TOF. The spectrum in (G) is after 3 hr with DTT added. (H) The extent of acetylation was quantified (n = 3 ± SD). All data are the mean ± SEM of at least three experiments unless otherwise stated. NS, not significant; ^∗^p < 0.05; ^∗∗^p < 0.01; ^∗∗∗∗^p < 0.0001. See also Figures S1 and S2.

To test whether AcCoA modified cysteine residues we included the thiol-alkylating reagent, *N*-ethyl maleimide (NEM; 25 mM). NEM reacts irreversibly with thiols and should block the transfer of the acetyl group of AcCoA to cysteines. Although NEM is selective for thiols, over long periods at pH 8, it also alkylates amines (Figures S1C and S1D) and can disrupt secondary structure (Figure S2A). Thus, we used two further thiol-blocking reagents, iodoacetamide (IAM; 25 mM) and methylmethanethiosulfonate (MMTS; 25 mM) that react more selectively with cysteine residues (Figures S1E and S1F). After 3 hr, 85%, 79%, and 83% of acetylation was prevented by NEM, IAM, and MMTS, respectively, suggesting substantial acetylation of cysteines by ^14^C-AcCoA (Figure 2A). To ensure NEM, IAM, and MMTS were not degrading AcCoA, we measured the change in AcCoA concentration by electrospray ionization (ESI) mass spectrometry (MS) (Figure 2B). After 3 hr, 70% of the AcCoA remained, and NEM, IAM, and MMTS did not accelerate AcCoA degradation. Thus, the lack of acetylation with NEM, IAM, and MMTS is not because exposure to AcCoA is diminished.

Consistent with acetylation of cysteine thiols, 71% of bound ^14^C could be removed by excess DTT (20 mM for 30 min), a broad specificity thiol reductant, after incubation with ^14^C-AcCoA (Figure 2C). Acetylation was reversed to a similar degree (67%) by hydroxylamine (HA; 50 mM for 30 min), a nucleophile with specificity for thioesters (Figure S2B), after the incubation with ^14^C-AcCoA (Figure 2C). Acetylation was insensitive to tris(2-carboxyethyl)phosphine (TCEP) (Figure 2C), a disulfide-reducing agent unreactive with thioesters (Figure S2B).

### Acetyl Moieties Migrate from Cysteine to Lysine Residues

Removing acetyl groups with DTT post-incubation was less effective at decreasing protein acetylation than blocking protein thiols with NEM, IAM, or MMTS preincubation, and the relative ineffectiveness of DTT increased with time (Figure S2C). Intramolecular *SN*-transfer reactions are often favorable and are used by enzymes such as acetyltransferases (Yan et al., 2002), as well as during native chemical ligation of peptides (Thapa et al., 2014). An increasing difference with time between preincubation with NEM, IAM, and MMTS and post-incubation with DTT (Figure S2C) could arise if AcCoA first *S*-acetylates a cysteine thiol through reversible thioester exchange and the acetyl moiety subsequently migrates in an irreversible intramolecular *SN*-transfer reaction to a nearby lysine. Consistent with this, preincubation of membranes with NEM, IAM, and MMTS all decreased lysine *N*-acetylation by western blot (Figures 2D, 2E, and S2D). In contrast to ^14^C-AcCoA (Figure 2C), post-incubation with DTT failed to remove the *N*-acetylation signal resulting from AcCoA.

To show this intramolecular *SN*-transfer reaction is a general proximity-based process that can occur on amino acid side chains without a favorable secondary structure that might bind AcCoA or stabilize nucleophiles, two peptides were synthesized that contained either a cysteine (CysPep) or a serine (SerPep) near a lysine residue (Figure 2F). Four deuterated standards were also made to facilitate quantification by MS (Figures S2E). When 200 μM of both peptides were simultaneously coincubated with 200 μM AcCoA in a competition assay, DTT-sensitive acetylation of CysPep rapidly increased, peaking at 20 min (Figure 2H). The initial rate of total acetylation of CysPep was ∼300-fold greater than that of SerPep, confirming the favorability of *S*-acetylation relative to direct *N*-acetylation. DTT-insensitive acetylation of CysPep (m/z = 1,122) continued to increase and was 23-fold higher (n = 3, p < 0.0001) than that of SerPep (m/z = 1,106) after 1 hr. At 3 hr, an additional DTT-insensitive peak (m/z = 1,164) appeared, consistent with CysPep acetylated on both lysine and serine (Figure 2G), and these modifications were confirmed by MALDI-TOF-TOF MS. Thus, initial reversible *S*-acetylation of a cysteine thiol via rapid thioester equilibration can facilitate subsequent *SN*-transfer of the acetyl moiety to a nearby lysine or serine residue. The rate of serine residue acetylation (pK_a_ ∼13) is expected to be slower than for lysine.

### GSH Alone Does Not Prevent *S*-acetylation

**Figure 3:**
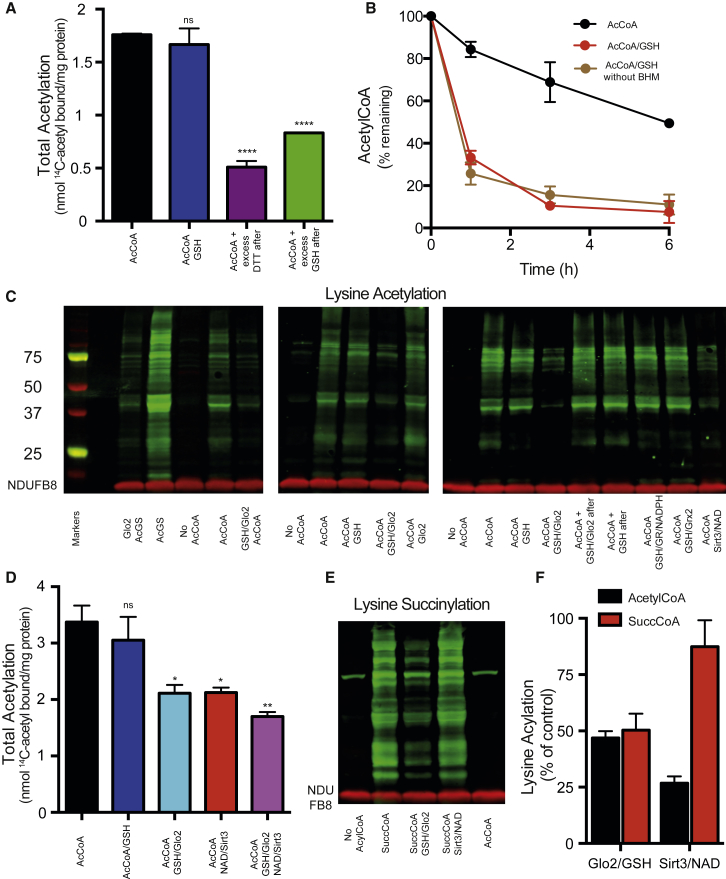
GSH and Glo2 Limit Acetylation (A) Acetylation is insensitive to the presence of GSH. Mitochondrial membranes were incubated with 2 mM ^14^C-AcCoA with or without 10 mM GSH at 37°C for 3 hr. At that point, an excess of DTT or GSH was added to some incubations for 30 min at 37°C. (B) GSH breaks down AcCoA. AcCoA (2 mM) was incubated with or without 10 mM GSH and with or without mitochondrial membranes at 37°C for up to 6 hr. AcCoA was measured by LC-MS/MS. Data are the mean ± range of two experiments. (C) *S*-acetylglutathione and AcCoA *N*-acetylate lysine residues are shown, and this is prevented by Glo2. Mitochondrial membranes were incubated with 2 mM AcCoA or 2 mM *S*-acetylglutathione at 37°C for 6 hr. This was supplemented with GSH (10 mM), NAD^+^ (1 mM), NADPH (1 mM), Glo2 (1 μg/mL), GR (0.1 μg/mL), Grx2 (1 μg/mL), or Sirt3 (10 μg/mL) during the 6 hr incubation. Excess GSH was then added for 30 min as indicated. After reducing SDS-PAGE, acetyllysine (green) and NDUFB8 (red) were visualized. (D) Acetylation by AcCoA is limited by Glo2 and Sirt3. Mitochondrial membranes were incubated with 2 mM ^14^C-AcCoA at 37°C for 6 hr. GSH (10 mM), Glo2 (1 μg/mL), NAD^+^ (1 mM), and Sirt3 (10 μg/mL) were added as indicated. (E and F) *N*-succinylation by succinylCoA (SuccCoA) is limited by Glo2, but not Sirt3. Mitochondrial membranes were incubated with 2 mM SuccCoA or AcCoA at 37°C for 6 hr. This was supplemented with GSH (10 mM), NAD^+^ (1 mM), Glo2 (1 μg/mL), or Sirt3 (10 μg/mL) during the 6 hr incubation as indicated. (E) After SDS-PAGE, succinyllysine (green) and NDUFB8 (red) were visualized. (F) The extent of lysine acetylation was quantified (n = 3 ± SD). All data are the mean ± SEM of at least three experiments unless otherwise stated. NS, not significant; ^∗^p < 0.05; ^∗∗^p < 0.01; ^∗∗∗∗^p < 0.0001. See also Figure S3.

High concentrations of the free thiol GSH could limit acetylation of protein thiols in vivo. Thus, we tested whether protein thiols could be acetylated in the presence of physiological concentrations of GSH (10 mM) and AcCoA (2 mM) and a physiological GSH-to-AcCoA ratio of 5 (Figure 2C). Surprisingly, 10 mM GSH during the 3 hr incubation with AcCoA only prevented 5% of the acetylation (Figure 3A). In contrast, an unphysiological 2,000-fold excess of GSH to protein thiols, added after the reaction of protein with AcCoA, removed 53% of the protein-bound acetyl moieties (Figure 3A), a level approaching DTT and HA (Figure 2C). Thus, *S*-acetylation of cysteine thiols still occurs with physiological concentrations of AcCoA and GSH but is reversible with excess GSH.

### Glo2 and GSH Limit Mitochondrial Protein Acetylation

Despite suggesting limited direct interaction between GSH and AcCoA, GSH caused a rapid decline in AcCoA (Figure 3B) and the appearance of a mass consistent with acetylglutathione (m/z = 348). This reaction did not require the presence of protein (Figure 3B) and is consistent with thioester equilibration between AcCoA and GSH. One explanation for why GSH did not prevent protein acetylation, despite depleting AcCoA, is that *S*-acetylglutathione (AcGS) also acetylates proteins. Incubation of mitochondrial membranes with 2 mM *S*-acetylglutathione (Figure 3C) led to *N*-acetylation of lysine residues increasing 44-fold. Although the pattern of protein acetylation was similar to that caused by AcCoA alone, the extent of lysine modification was 3.1 ± 0.2-fold higher. Acetylation by *S*-acetylglutathione was also sensitive to NEM, IAM, and MMTS (Figure S3A). There was no acetylation by *O*-linked acetylcarnitine (Figure S3B).

*S*-acetylglutathione is a good substrate for Glo2, an enzyme that hydrolyses a range of thioesters conjugated to GSH (Talesa et al., 1989). Glo2 catalyzes the second stage of the two-step detoxification pathway for glyoxals that form during glycolysis in the cytoplasm. Glyoxalase I (Glo1) conjugates these dicarbonyls to GSH via a thioester bond, and Glo2 subsequently recognizes the GSH moiety and cleaves this thioester. Mitochondria contain Glo2, but not Glo1, suggesting that mitochondrial Glo2 has an alternate role (Rabbani et al., 2014). When human Glo2 (20 ng, 0.5 mU) was added to an incubation of *S*-acetylglutathione and mitochondrial membranes, *N*-acetylation decreased by 68% ± 9% (Figure 3C). Next, we tested whether *N*-acetylation by 2 mM AcCoA was also prevented by Glo2 (Figures 3C and S3B). When GSH was combined with Glo2, *N*-acetylation decreased by 54% ± 4% (n = 7, p < 0.0001). However, neither GSH nor Glo2 alone significantly decreased *N*-acetylation by AcCoA (Figures 3C and S3C).

Mitochondria contain mechanisms to recycle disulfides that might also act on *S*-acetylglutathione or acetylated cysteine residues. GSH reductase (GR) reduces glutathione disulfide (GSSG) using NADPH, and the addition of yeast GR (2 ng, 0.44 mU), 1 mM NAPDH, and 10 mM GSH did not decrease protein *N*-acetylation (Figure 3C). Glutaredoxin 2 (Grx2) reduces protein-GSH mixed disulfides in the presence of GSH, and the addition of human Grx2 (20 ng, 0.4 mU) with 10 mM GSH also failed to decrease protein *N*-acetylation (Figure 3C). Protein disulfides can be reduced by thioredoxin reductase (TR) and thioredoxin (Trx), but the addition of TR (20 ng, 0.4 mU), Trx (5 μM), and 1 mM NADPH had no effect on protein acetylation (Figure S3D). Mitochondria contain Sirt3, which can remove acetyl groups from lysine residues. As expected, human recombinant Sirt3 (200 ng, 0.0004 mU) with 1 mM NAD^+^ reversed protein *N*-acetylation by AcCoA by 70% ± 4% (Figure 3C).

Next, we measured whether total protein acetylation by ^14^C-AcCoA was sensitive to GSH/Glo2 or NAD^+^/Sirt3 alone or in combination. Incubation with 2 mM AcCoA for 6 hr led to protein acetylation, and this was decreased 37% ± 2% or 37% ± 3% by the presence of GSH/Glo2 or NAD^+^/Sirt3, respectively (Figure 3D). Glo2 and Sirt3 in combination decreased acetylation by 53% ± 3%.

### Glo2 Can Limit Acylation by Other AcCoA Species

Although AcCoA is the most common form of activated CoA in vivo, it is not the only AcCoA present. CoA is used extensively as a cofactor in metabolism, and many other moieties are conjugated to its thiol, e.g., succinylCoA in the tricarboxylic acid cycle. One potential advantage of GSH/Glo2 over NAD^+^/Sirt3 is that Glo2 is selective for GSH, but not the acyl moiety conjugated to its thiol (Talesa et al., 1989, Vander Jagt, 1993). To test whether Glo2 could diminish succinylation, we incubated mitochondrial membranes with 2 mM succinylCoA. GSH/Glo2, but not NAD^+^/Sirt3, could decrease lysine succinylation (Figures 3E and 3F). Thus, GSH and Glo2 provide a broad specificity mechanism for removing AcCoA-dependent modifications on cysteine residues and consequently limiting their accumulation on lysine residues.

## Discussion

Although reactions between AcCoA and cysteine thiols have been noted previously (Bizzozero et al., 2001), almost all acetylation literature focuses on *N*-acetylation of lysine residues by acetyltransferase enzymes (McDonnell et al., 2015). It has been recognized that *N*-acetylation can also occur non-enzymatically (Wagner and Payne, 2013, Wagner and Hirschey, 2014, Weinert et al., 2015, Davies et al., 2016). Our results show that non-enzymatic *N*-acetylation frequently occurs indirectly via the reversible *S*-acetylation of a nearby thiol. This thioester exchange, followed by an intramolecular *SN*-transfer reaction to a proximal amine, underlies the native chemical ligation of peptides (Thapa et al., 2014), autoacetylation of tau protein (Cohen et al., 2013), and reactions in the active sites of enzymes such as acetyltransferases (Yan et al., 2002, Cohen et al., 2013). Here we show that this intramolecular *SN*-transfer reaction is likely to be widespread, because it proceeds rapidly on a peptide lacking secondary structure (Figures 2G and 2H). Although secondary structure is not a requirement (Figure 2H) and the apparent K_M_ of the reaction is high (Figure 1C), we would expect enhancement of lysine acetylation, in which the secondary structure stabilizes a nucleophile or leads to AcCoA associating with a protein. AcCoA shares an ADP-based backbone with ATP, NAD(P)H, and flavin adenine dinucleotide (FADH), and Rossmann-fold binding sites for these cofactors are prevalent throughout the cell.

**Figure 4:**
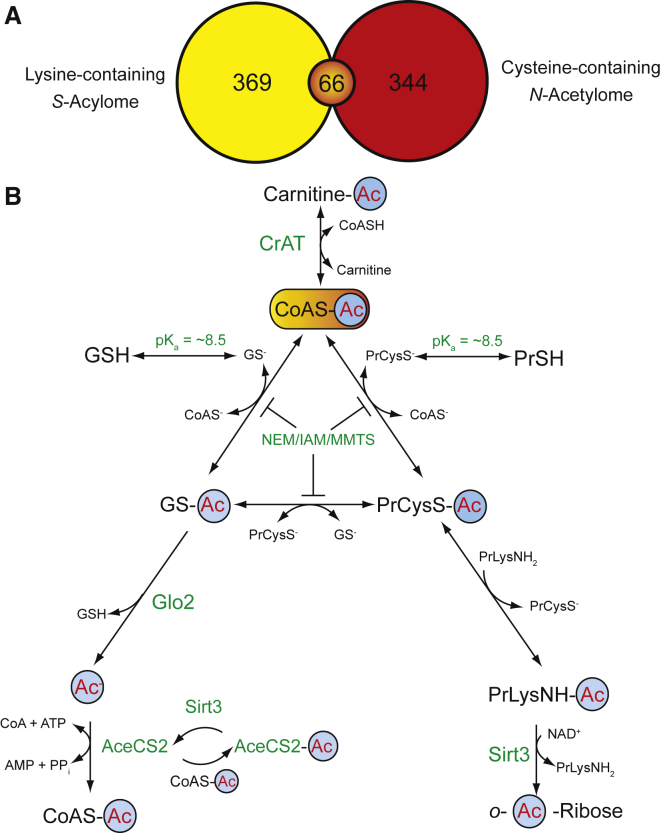
Model of Thiol Involvement in Non-enzymatic Protein Acetylation (A) Coexistence of *N*-acetyllysine and *S*-acylcysteine. Overlap (orange) between lysine-containing peptides in the mouse liver *S*-acylome (yellow) (Gould et al., 2015) and cysteine-containing peptides (red) in the mouse liver mitochondrial *N*-acetylome (blue) (Rardin et al., 2013). (B) Model of non-enzymatic acetylation. The steady-state concentration of reactive AcCoA is buffered lower by carnitine and carnitine acetyltransferase (CrAT). The pK_a_ and relative concentrations of GSH and protein thiols (PrCysSH) determine the rate at which AcCoA reacts with their thiolate forms, GS^−^ and PrCysS^−^. NEM, IAM, and MMTS limit reversible thioester exchange by blocking thiols. Glo2 shifts the equilibrium away from cysteine-bound acetyl groups (PrCysSAc) by hydrolyzing *S*-acetylglutathione to acetate (Ac^−^). PrCysSAc not removed by GSH can irreversibly transfer to a nearby lysine (PrLysNH_2_), where Sirt3 can degrade it. AcCoA is regenerated by AcCoA synthetase (AceCS2), and this is regulated by *N*-acetylation and Sirt3. See also Figure S4 and Table S1.

Our use of HA to selectively cleave the thioester bond was based on its widespread use to study *S*-acylation-dependent membrane anchoring by *S*-acyltransferases and palmitoylCoA (Roth et al., 2006). Consequently, some of the 627 *S*-acylcysteine residues identified by MS in a mouse liver *S*-acylation dataset could have been *S*-acetylated by AcCoA (Gould et al., 2015). Merging this dataset with the 344 acetyllysines on peptides that also contain a cysteine residue in a mouse liver mitochondrial acetylome (Rardin et al., 2013) shows 66 acetyllysine modifications on peptides, which also contained a *S*-acylated cysteine residue (Figure 4A; Table S1). Thus, a number of acetylcysteine and acetyllysine residues coexist in vivo and may be worthy of further study.

Enrichment with anti-acetyllysine antibodies and improvements in MS have identified an increasing number of lysine acetylation sites (Rardin et al., 2013). However, purification of acetylated from unacetylated peptides limits understanding of stoichiometry and physiological importance (Weinert et al., 2015). Bovine heart mitochondrial membranes contain 53 nmol of exposed thiols per milligram of protein (Requejo et al., 2010), and 0.78% of exposed cysteine thiols were acetylated every hour by 2 mM AcCoA (see Supplemental Information). This corresponds to 0.13% of exposed lysine residues *N*-acetylated each hour. Our in vitro concentration of exposed thiols is ∼265 μM, which is ∼100-fold lower than in vivo; consequently, the rate of the reaction between AcCoA and protein could be much higher. However, basal *N*-acetylation of the mitochondrial membrane preparation, a function of acetylation and deacetylation, is 17% of that after 6 hr with 2 mM AcCoA (Figure 2F). This is equivalent to 0.13% of exposed lysine residues, a value consistent with the median *N*-acetylation stoichiometry of liver mitochondrial proteins (0.11%) observed by MS (Weinert et al., 2015). Thus, it is likely, at least in mitochondria, that most acetylome arises from low-level non-enzymatic acetylation.

The low stoichiometry of residue modification suggests this process is analogous to oxidative stress, in which reactive oxygen species (ROS) non-enzymatically modify protein, DNA, and lipid. One striking difference is that AcCoA is central to metabolism, so it cannot be directly degraded. This limits protective strategies to preventing excess AcCoA and removal of acetyl groups from proteins when they form. In addition to Sirt3, it has been shown that carnitine acetyltransferase (CrAT) buffers the AcCoA concentration by storing excess AcCoA as unreactive *O*-acetylcarnitine (Figure S3B), thus limiting the thioester concentration and consequently cysteine and lysine acetylation (Davies et al., 2016). Here we identify another system capable of limiting CoA-derived modifications: the mitochondrial GSH and Glo2 system (Figure 4B). Cytosolic Glo2 and Glo1 detoxify glyoxals formed during glycolysis; however, an alternate function is required for mitochondrial Glo2, because the matrix lacks glycolysis and Glo1 (Rabbani et al., 2014). We suggest that a major function of matrix Glo2 is to degrade *S*-acetylglutathione and thereby shift the equilibrium away from cysteine *S*-acetylation, thus limiting acetylation of vital cysteine and lysine residues (Figure 4B). GSH at physiological concentrations is ineffective, because the product of the reaction is *S*-acetylglutathione, which could simply reacetylate protein, as thioester exchange is faster than non-enzymatic hydrolysis (Bracher et al., 2011). Glo2 is advantageous, because it increases the rate of hydrolysis of a range of acylglutathione species (Talesa et al., 1989) without affecting their corresponding AcCoAs.

Enzyme active sites have evolved over time to be efficient, and many have intermediate steps with substrate covalently linked to active site residues. We suspect protein modifications can result from unintentional chemistry on less evolved protein surfaces, where the second step releasing the modification is absent or the target is a proximal amino acid. The AcCoA concentration is ∼1,000-fold greater than most ROS and AcCoA cannot be scavenged; together this represents both a problem for protein function and an opportunity for regulation. Perhaps these regulatory pathways arose because the enzymes required to switch them off, sirtuins, had already evolved to prevent non-specific damage to protein.

Although many of the low-occupancy, potentially deleterious acetylation sites identified by MS may arise from the *SN*-transfer described here, highly acetylated regulatory proteins may use a combination of features to achieve a robust desirable signal. Consequently, the relative contribution of proximal cysteine thiols within signaling pathways would need to be explored case by case with cysteine mutations. Furthermore, although this work provides a potential explanation for the expression of Glo2 in mitochondria, the physiological relevance of *S*-acylation is still unknown and will need to be determined in vivo by the use of knockout animals.

## Experimental Procedures

### Materials

Recombinant human Glo2 (25 nmol/min/μg) and Sirt3 (2 pmol/min/μg) were from R&D Systems. Rabbit anti-acetyllysine (9441) was from Cell Signaling Technology. Rabbit anti-succinyllysine (PTM-401) was from PTM Biolabs. Bovine heart mitochondrial membrane fragments were prepared as described previously and stored at −80°C until used (Sharpley et al., 2006).

### Radioactive Acetylation of Protein

Mitochondrial membrane fragments (5 mg/mL) were suspended in 20 μL of KP_i_ buffer (50 mM KH_2_PO_4_, 100 μM EDTA, 100 μM diethylenetriaminepentaacetic acid (DTPA) [pH 7.8]) supplemented with NEM (25 mM), IAM (25 mM), MMTS (25 mM), human Glo2 (1 μg/mL), human Sirt3 (10 μg/mL), NAD^+^ (1 mM) or GSH (10 mM) as indicated. When NEM, IAM, and MMTS were used, they were preincubated with mitochondrial membrane fragments for 15 min at 37°C before the addition of AcCoA. The reaction was started with 50 μM ^14^C-AcCoA and a 2 mM mixture of non-radioactive AcCoA and CoA before incubation for up to 6 hr at either 0°C or 37°C. The reaction was quenched by the addition of 980 μL of KP_i_ buffer. To this 1 mL volume, a large excess of DTT (20 mM), HA (50 mM), TCEP (20 mM), or GSH (20 mM) was sometimes added, followed by a further 30 min incubation at 37°C. Samples were spun at 16,000 × *g* for 10 min with the supernatant discarded and the pellet washed with a further 1 mL of 50 mM KP_i_ buffer. The supernatant was discarded, and the inside of the tube dried. The pellet was resuspended in 50 μL of 20% (v/v) Triton X-100. When ^14^C-AcCoA was added after the incubation, ^14^C counts in the pellet water space were ∼5% of the total at 6 hr. EDTA and DTPA were omitted from the buffer for experiments with Glo2 (1 μg/mL) and Sirt3 (10 μg/mL). The specific activity of recombinant Sirt3 is much lower than recombinant Glo2, hence the addition of ten times as much protein.

### Lysine Acetylation

Mitochondrial membrane fragments (5 mg/mL) were suspended in 20 μL of NaP_i_ buffer (50 mM NaH_2_PO_4_, 100 μM EDTA, 100 μM DTPA [pH 7.8]) supplemented with NEM (25 mM), IAM (25 mM), MMTS (25 mM), human Glo2 (1 μg/mL), human Sirt3 (10 μg/mL), human Grx2 (1 μg/mL), yeast GR (0.1 μg/mL), *E. coli* TR (1 μg/mL), *E. coli* Trx (5 μM), NAD^+^ (1 mM), NADPH (1 mM), or GSH (10 mM) as indicated. The reaction was started with 2 mM AcCoA or 2 mM *S*-acetylglutathione. When NEM, IAM, and MMTS were used, they were preincubated for 15 min at 37°C before the addition of AcCoA or *S*-acetylglutathione and then incubated for a further 3 or 6 hr at 37°C. The samples were mixed 1:1 with loading buffer containing 200 mM DTT before being run on a 12% SDS-PAGE gel. This DTT in the loading buffer reduces disulfides such as those generated by MMTS and remove cysteine-bound acetyl groups. Because the MMTS adduct is reduced by DTT in the loading buffer, the loss of signal does not result from an altered antigen-antibody interaction.

### Small-Molecule ESI MS

20 μL of fresh HCO_3_^−^ buffer (50 mM NH_4_HCO_3_ [pH 7.8]) were supplemented with mitochondrial membrane fragments (5 mg/mL), AcCoA (2 mM), GSH (10 mM), GSSG (5 mM), NEM (25 mM), IAM (25 mM), and MMTS (25 mM) as indicated. They were then incubated for up to 6 hr at 37°C before the addition of 980 μL of HCO_3_^−^ buffer to quench the reaction. The samples were spun at 16,000 × *g* for 2 min, and the filtered supernatant was directly infused into a Xevo TQ-S triple quad mass spectrometer (Waters) at 50 μL/min. Samples were assessed using ESI in negative mode.

### MALDI of Synthetic Peptides

Two synthetic peptides were designed, butyryl-RYAKGCASR-NH_2_ (CysPep) and butyryl-RYAKGSASR-NH_2_ (SerPep). The following features were designed into the synthetic peptides: one tyrosine for quantification by UV, one arginine at each end to facilitate MS of ion fragments, and a glycine between the lysine and the cysteine or serine for flexibility. The peptides also contained a butyrated N-terminal amide and a C-terminal amide, a common approach to improve stability and remove terminal charges that would not be present on most peptides within an intact protein. Four additional deuterated synthetic peptides were made as standards using deuterated butyric acid: d7-butyryl-RYAKGCASR-NH_2_ (d7-CysPep), d7-butyryl-RYAKGSASR-NH_2_ (d7-SerPep), d7-butyryl-RYAK_Ac_GCASR-NH_2_ (d7-AcCysPep), and d7-butyryl-RYAK_Ac_GSASR-NH_2_ (d7-AcSerPep). For the reaction, 200 μM cysteine peptide, 200 μM serine peptide, 200 μM fresh AcCoA, and 1 mM fresh TCEP were coincubated together in 25 μL of fresh 10 mM NH_4_HCO_3_ (pH 7.8) at 37°C. After the incubation, a 10 μL aliquot of the reaction was added to 10 μL of either 40 mM DTT or H_2_O. The DTT sample was incubated for a further 30 min at 37°C. To both aliquots, 475 μL of 0.1% trifluoroacetic acid (TFA) was added to quench further nucleophilic reactions, followed by 5 μL of a deuterated standard mix (50 μM d7-CysPep, 50 μM d7-SerPep, 50 μM d7-AcCysPep, and 50 μM d7-AcSerPep). For the 0 hr time point, AcCoA was added after 0.1% TFA.

The sample was spotted on the MALDI plate using the bottom-layer method. In total, 20 spectra with ten shots each were collected per spot, using a minimum intensity of 1,000 and a maximum intensity of 10,000 as the selection criteria. Peak intensities from three spots per experiment were quantified using mMass, and the concentration was calculated using summed intensities of the peptides and the d7 standards. Data are the average ± SEM of three experiments on separate days, each with three spots.

### Statistics and Data Processing

Statistical significance was determined, usually relative to incubation with AcCoA, using a two-tailed Student’s t test or one-way ANOVA followed by a Dunnett’s multiple comparison test in Prism v.6. The overlap of the *S*-acylation (Gould et al., 2015) and *N*-acetylation (Rardin et al., 2013) datasets was determined using pgAdmin3. The peptides in the *N*-acetylation dataset contain two neighboring tryptic peptides joined together, because trypsin does not cleave at acetyllysine. Overlap occurred if either part of this miscleaved peptide was also in the *S*-acylation dataset.

See Supplemental Information for further details.

## Author Contributions

A.M.J. performed most of the experiments and data analysis and prepared the manuscript with M.P.M. M.P.M. was the grant holder. K.H. synthesized the peptides. A.R.H. supplied tissue. K.H. and I.M.F. gave suggestions about chemistry and MS, respectively. K.H., A.L., and S.D. processed samples for MS.

## Supplemental Information

Document S1. Supplemental Experimental Procedures, Figures S1–S3

Table S1. Overlap of Cysteine Acylation and Lysine Acetylation, Related to Figure 4

Document S2. Article plus Supplemental Information
