# Supplementary material for: Non-enzymatic N-acetylation of Lysine Residues by AcetylCoA Often Occurs via a Proximal S-acetylated Thiol Intermediate Sensitive to Glyoxalase II
Source: Cell Rep. 2017 Feb 28;18(9):2105–12. doi: 10.1016/j.celrep.2017.02.018 (PMC6381604; doi:10.1016/j.celrep.2017.02.018)
Supplement: Document S1. Supplemental Experimental Procedures, Figures S1–S3 [file mmc1.pdf]

Cell Reports, Volume *18*

Supplemental Information

Non-enzymatic *N*-acetylation of Lysine Residues by AcetylCoA Often Occurs via a Proximal *S*-acetylated Thiol Intermediate Sensitive to Glyoxalase II

Andrew M. James, Kurt Hoogewijs, Angela Logan, Andrew R. Hall, Shujing Ding, Ian M. Fearnley, and Michael P. Murphy

Supplementary Materials

Non-enzymatic *N*-Acetylation of Lysine Residues by AcetylCoA Often Occurs via a Proximal *S*-Acetylated Thiol Intermediate Sensitive to Glyoxalase II

Andrew M. James[1,*], Kurt Hoogewijs[1,2,3], Angela Logan[1], Andrew R. Hall[1], Shujing Ding[1], Ian M. Fernley[1] and Michael P. Murphy[1,*,§]

[1]Medical Research Council Mitochondrial Biology Unit, Cambridge, CB2 0XY, UK

[2]Medical Research Council Laboratory of Molecular Biology, Cambridge, CB2 0QH, UK

[3]The Wellcome Trust Centre for Mitochondrial Research, Institute for Cell and Molecular Biosciences, The Medical School, Newcastle University, Newcastle upon Tyne, NE2 4HH, UK

[*]Correspondence: aj@mrc-mbu.cam.ac.uk; mpm@mrc-mbu.cam.ac.uk

SUPPLEMENTAL CALCULATIONS

Bovine heart mitochondrial membranes contain 53 nmol of exposed thiols/mg protein (Requejo et al., 2010). After 3h of incubation with 2 mM AcCoA, 1.75 nmol of acetyl was bound per mg of protein and of this 71% was sensitive to DTT and bound to cysteine (Figure 3A).

1.75 / 53 * 0.71 / 3 = 0.78% of exposed cysteines acetylated per h *in vitro* under these conditions

The frequency of lysine within protein (5.8%) is ~3-fold higher than cysteine (1.8%) (Trinquier and Sanejouand, 1998) and of these 4% of lysines and 47% of cysteines are buried (Schein, 1990). As we observed 29% of acetylation was DTT-insensitive (Figure 3A) it can be estimated that ~0.13% of exposed lysine residues are *N*-acetylated each hour.

0.78 / (5.8 / 1.8) * (0.96 / 0.53) * 0.29 = 0.13% exposed lysines acetylated per h *in vitro* under these conditions

Basal *N*-acetylation of the bovine heart mitochondrial membrane preparation is 17% of that observed after 6 h with 2 mM AcCoA (Figure 2F). This is equivalent to ~0.077% of exposed lysine residues.

0.13 * 6 * 0.17 = 0.13% exposed lysine amines basally acetylated *in vivo*

SUPPLEMENTAL EXPERIMENTAL PROCEEDURES

Materials – Recombinant human Glo2 (25 nmol/min/µg) and Sirt3 (2 pmol/min/µg) were from R&D Systems (Minneapolis, USA). ^{14}C-AcCoA was from American Radiolabelled Chemicals (St. Loius, USA). ^{14}H-GSH was from Perkin Elmer (Waltham, USA). ^{14}H-GSSG was prepared by mixing 500 nM ^{14}H-GSH with 25 mM GSSG for 1 h at 37˚C at pH 7.8. *S*-acetylglutathione (AcGS) was from Iris Biotech (Marktredwitz, Germany). Rabbit anti-acetyllysine (9441) was from Cell Signaling Technology (Danvers, USA). Rabbit anti-succinyllysine (PTM-401) was from PTM Biolabs (Chicago, USA). Mouse anti-NDUFB8 (ab110242) was from Abcam (Cambridge, UK). Anti-mouse and anti-rabbit fluorescent secondaries were from LI-COR Biosciences (Lincoln, USA). All other enzymes and chemicals were from Sigma. Bovine heart mitochondrial membrane fragments were prepared as described previously and stored at -80˚C until used (Sharpley et al., 2006). The bovine heart mitochondrial membranes were diluted 10-fold in appropriate buffer (see below), pelleted at 16,000 x g for 5 min and resuspended at 10 mg/mL in appropriate buffer to remove Tris. Solutions of TCEP, HA, GSH, GSSG, and AcGS were made up in buffer and neutralised prior to use.

Radioactive acetylation of protein - Bovine heart mitochondrial membrane fragments (5 mg/mL) were suspended in 20 µL of KP_i buffer (50 mM KH_2PO_4, 100 µM EDTA, 100 µM DTPA, pH 7.8) supplemented with *N*-ethyl maleimide (NEM; 25 mM), iodoacetamide (IAM; 25 mM), methylmethanethiosulphonate (MMTS; 25 mM), human Glo2 (1 µg/mL), human Sirt3 (10 µg/mL), 1 mM NAD^+ or 10 mM GSH as indicated. When NEM, IAM and MMTS were used they were preincubated with bovine heart mitochondrial membranes for 15 min at 37˚C prior to addition of AcCoA. The reaction was started with 50 µM ^{14}C-AcCoA and either 2 mM cold AcCoA or CoA and then they were incubated for up to 6h at either 0˚C or 37˚C. The reaction was quenched by the addition of 980 µL of KP_i buffer. To this 1 mL volume a large excess of dithiothreitol (DTT; 20 mM), hydroxylamine (HA; 50 mM), TCEP (20 mM) or GSH (20 mM) were sometimes added followed by a further 30 min incubation at 37˚C. Samples were spun at 16,000 x g for 10 min with the supernatant discarded and the pellet washed with a further 1 mL of 50 mM KP_i buffer. The supernatant was discarded and the inside of the tube dried with tissue. The pellet was resuspended in 50 µL of 20% (v/v) Triton X-100.

To control for unbound AcCoA in the pellet water space, 50 µM ^{14}C-AcCoA was omitted from the incubation and instead added immediately to the 1 mL of 50 mM KP_i buffer used to quench the reaction and before centrifugation. When ^{14}C-AcCoA was added after the incubation ^{14}C counts in the pellet water space were ~5% of the total at 6 h. EDTA and DTPA were omitted from the buffer for experiments with Glo2 (1 µg/mL) and Sirt3 (10 µg/mL). The specific activity of recombinant Sirt3 is much lower than recombinant Glo2, hence the addition of ten times as much protein.

Lysine acetylation - Bovine heart mitochondrial membrane fragments (5 mg/mL) were suspended in 20 μL of NaP_i buffer (50 mM NaH_2PO_4, 100 μM EDTA, 100 μM DTPA, pH 7.8) supplemented with NEM (25 mM), IAM (25 mM), MMTS (25 mM), human Glo2 (1 μg/mL), human Sirt3 (10 μg/mL), human Grx2 (1 μg/mL), yeast GR (0.1 μg/mL), *E.coli* TR (1 μg/mL), *E.coli* Trx (5 μM), 1 mM NAD^+, 1 mM NADPH or GSH (10 mM) as indicated. The reaction was started with 2 mM AcCoA or 2 mM *S*-acetyl-glutathione (AcGS). When NEM, IAM and MMTS were used they were preincubated for 15 min at 37˚C prior to addition of AcCoA or AcGS and then they were then incubated for 3 or 6h at 37˚C. The samples were mixed 1:1 with loading buffer containing 200 mM DTT before being run on a 12% SDS-PAGE gel. This DTT in the loading buffer will reduce disulphides such as those generated by MMTS and remove cysteine-bound acetyl groups. As the MMTS adduct is reduced by DTT in the loading buffer the loss of signal does not result from an altered antigen-antibody interaction. They were transferred to PVDF and blocked overnight at 4˚C with Odyssey® Blocking Buffer. The PVDF membrane was probed with 1/1000 rabbit anti-acetyllysine or rabbit anti-succinyllysine and 1/5000 mouse anti-NDUFB8 then visualised with anti-mouse and anti-rabbit fluorescent secondaries at 680 nm and 800 nm, respectively. Fluorescence intensity was measured using a LI-COR Odyssey® CLx near-infrared imaging system and Image Studio v4.0. EDTA and DTPA had no appreciable effect and were omitted for experiments with Glo2 (1 μg/mL) and Sirt3 (10 μg/mL).

Complex I-dependent NADH:hexaammineruthenium (HAR) activity - Bovine heart mitochondrial membrane fragments (5 mg/mL) were suspended in 20 μL of 50 mM NaH_2PO_4 (pH 7.8) supplemented with NEM (25 mM), IAM (25 mM), MMTS (25 mM). After incubation at 37˚C, 2 μL samples were taken and added to 18 μL of 20 mM Tris (pH 7.5) and snap frozen. After thawing, 5 μL of each diluted sample was added to per well of a 96-well plate. To this was added 200 μL of 200 μM NADH and 3.5 mM HAR in 20 mM Tris (pH 7.5) containing 4 mg/mL rotenone. The rate of NADH loss was measured at 340-380 nm using a Molecular Devices SpectraMax Plus 384 platereader. Unlike Complex I dependent NADH:Q activity, NADH:NAR activity is insensitive to alkylating reagents and rotenone, consequently loss of activity likely represents Complex I denaturation.

Small molecule ESI mass spectrometry - 20 μL of fresh bicarbonate buffer (50 mM NH_4HCO_3, pH 7.8) supplemented with bovine heart mitochondrial membranes (5 mg/mL), AcCoA (2 mM), GSH (10 mM), GSSG (5 mM), NEM (25 mM), IAM (25 mM), and MMTS (25 mM) as indicated. They were then incubated for up to 6h at 37˚C before the addition of 980 μL of bicarbonate buffer to quench the reaction. The samples were spun at 16,000 x g for 2 min and the filtered supernatant was directly infused into a Xevo TQ-S triple quad mass spectrometer (Waters) at 50 μL/min. Samples were assessed using electrospray ionisation in negative mode, with nitrogen as the curtain gas. Instrument parameters were: capillary voltage 3.1 kV; cone voltage 25 V; ion source temperature 150˚C. Data were acquired for one minute from 50 – 1200 m/z and total ion counts assessed using Masslynx software (Waters).

MALDI of synthetic peptides - Two synthetic peptides were designed, butyryl-RYAKGCASR-NH_2 (CysPep) and butyryl-RYAKGSASR-NH_2 (SerPep). The following features were designed into the synthetic peptides, one tyrosine for quantification by UV, one arginine at each end to facilitate MS of ion fragments, a glycine between the lysine and cysteine/serine for flexibility. The peptides also contained a butyrated N-terminal and a C-terminal amide, a common approach to improve stability and remove terminal charges that would not be present on most peptides within an intact protein. Peptides were synthesised manually on Rink amide ChemMatrix using Fmoc chemistry with 5 equivalents of Fmoc-amino acids or butyric acid, and PyBOP and DIPEA as coupling reagents. The obtained peptides were cleaved off the resin using TFA/DODT/TIS/H_2O (94:2.5:1:2.5), precipitated with diethyl ether and purified on HPLC (Waters XBridge Peptide BEH C18 OBD Prep Column, 300Å, 5 μm, 19 mm X 50 mm, 12 ml/min, from 100% water + 0.1% TFA to 100% Acetonitrile + 0.1% TFA). Fractions with purity >95% were collected and lyophilised. Four additional deuterated synthetic peptides were made as standards using deuterated butyric acid, d7-butyryl-RYAKGCASR-NH_2 (d7-CysPep), d7-butyryl-RYAKGSASR-NH_2 (d7-SerPep), d7-butyryl-RYAK_{Ac}GCASR-NH_2 (d7-AcCysPep) and d7-butyryl-RYAK_{Ac}GSASR-NH_2 (d7-AcSerPep). Trifluoroacetic (TFA) salts of the peptides were dissolved in water at ~1 mM then quantified by UV using tyrosine absorbance at 280 nm (1.49 $mM^{-1}.cm^{-1}$) and diluted to 500 μM in water. The peptides were stored like this at acidic pH to limit cysteine oxidation. For the reaction 200 μM cysteine peptide, 200 μM serine peptide, 200 μM fresh AcCoA and 1 mM fresh TCEP were coincubated in 25 μL fresh

bicarbonate buffer (10 mM NH_4HCO_3, pH 7.8) at 37˚C. After the incubation a 10 μL aliquot of the reaction was added to 10 μL of either 40 mM DTT or H_2O. The DTT sample was incubated for a further 30 min at 37˚C. To both aliquots 475 μL 0.1% TFA was added to quench further nucleophilic reactions followed by 5 μL of a deuterated standard mix (50 μM d7-cysteine peptide, 50 μM d7-serine peptide, 50 μM d7-acetylated cysteine peptide and 50 μM d7-acetylated serine peptide). For the 0 h time point, AcCoA was added after 0.1% TFA.

The sample was spotted on the MALDI-plate using the bottom-layer method. Matrix (0.75μL, 50% acetonitrile, 5 mg/ml α-cyano-4-hydroxycinnamic acid, 10 mM dibasic ammonium citrate, 0.1% TFA) was spotted on the plate and 0.75 μL sample was mixed in. The spot was left to dry at RT, after which another layer of 0.75 μL of matrix was added. In total 20 spectra with 10 shots each were collected per spot, using a minimum intensity of 1000 and a maximum of 10000 as selection criterion. Peak intensities from 3 spots per experiment were quantified using mMass and the concentration calculated using summed intensities of the peptides and the d7 standards. Data is the average ± SEM of 3 experiments on separate days each with 3 spots.

Statistics and data processing – Statistical significance was determined, usually relative to incubation with AcCoA, using a two-tailed Student's t-test or one-way ANOVA followed by a Dunnett's multiple comparison test in Prism v6. The overlap of the *S*-acylation (Gould et al., 2015) and *N*-acetylation (Rardin et al., 2013) datasets was determined using pgAdmin3. The peptides in the *N*-acetylation dataset contain two neighbouring tryptic peptides joined together as trypsin does not cleave at acetyllysine. Overlap occurred if either part of this miscleaved peptide was also in the *S*-acylation dataset.

SUPPLEMENTAL REFERENCES

Schein, C. H. 1990. Solubility as a function of protein structure and solvent components. *BioTechnology,* 8**,** 308-317.

Sharpley, M. S., Shannon, R. J., Draghi, F. & Hirst, J. 2006. Interactions between phospholipids and nadh:Ubiquinone oxidoreductase (complex i) from bovine mitochondria. *Biochemistry,* 45**,** 241-8.

Trinquier, G. & Sanejouand, Y. H. 1998. Which effective property of amino acids is best preserved by the genetic code? *Protein Eng,* 11**,** 153-69.

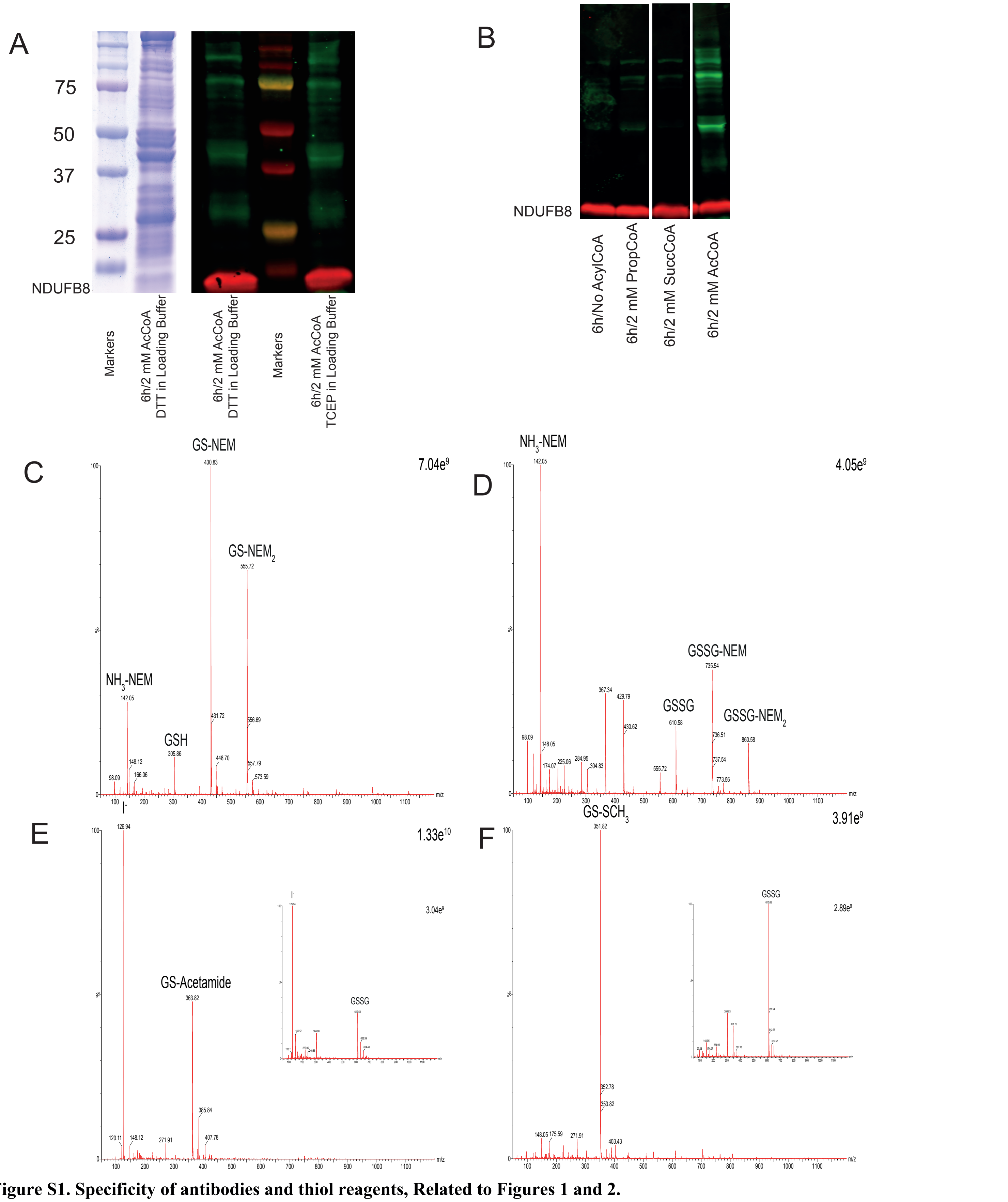

Figure S1. Specificity of antibodies and thiol reagents, Related to Figures 1 and 2.

A, the anti-acetyllysine antibody does not recognise *S*-acetylcysteine. Bovine heart mitochondrial membranes (5 mg/mL) were incubated with 2 mM AcCoA at 37˚C for 6 h. Samples were run on SDS-PAGE containing either 5 mM TCEP or 5 mM DTT. After SDS-PAGE total protein was visualised with coomassie or acetyllysine (green) and NDUFB8 (red) were visualised with fluorescent secondaries. B, the anti-acetyllysine antibody does not recognise propionyllysine or succinyllysine. Bovine heart mitochondrial membranes (5 mg/mL) were incubated with 2 mM propionyl-CoA (PropCoA), succinyl-CoA (SuccCoA) or AcCoA at 37˚C for 6 h. Samples were run on SDS-PAGE with 100 mM DTT. After SDS-PAGE acetyllysine (green) and NDUFB8 (red) were visualised with fluorescent secondaries. C-F, reactivity of NEM, IAM and MMTS with amines and thiols. GSH (10 mM) or GSSG (5 mM) were incubated in 50 mM NH_4HCO_3 (pH 7.8) buffer supplemented with 25 mM NEM, IAM or MMTS for 3 h at 37˚C. The sample was diluted 50-fold with NH_4HCO_3 (pH 7.8) buffer and GSH adducts were visualised by ESI MS in negative-ion mode. A and B, NEM also reacts with amines. NEM alkylates GSH and GSSG in two places, consistent with alkylation of one thiol and one amine on GSH or two amines on GSSG. It also alkylates buffer NH_3. C, IAM does not react with amines. IAM alkylates GSH in one place and does not alkylate GSSG (inset), consistent with alkylation of one thiol. D, MMTS does not react with amines. MMTS alkylates GSH in one place and does not alkylate GSSG (inset), consistent with alkylation of one thiol.

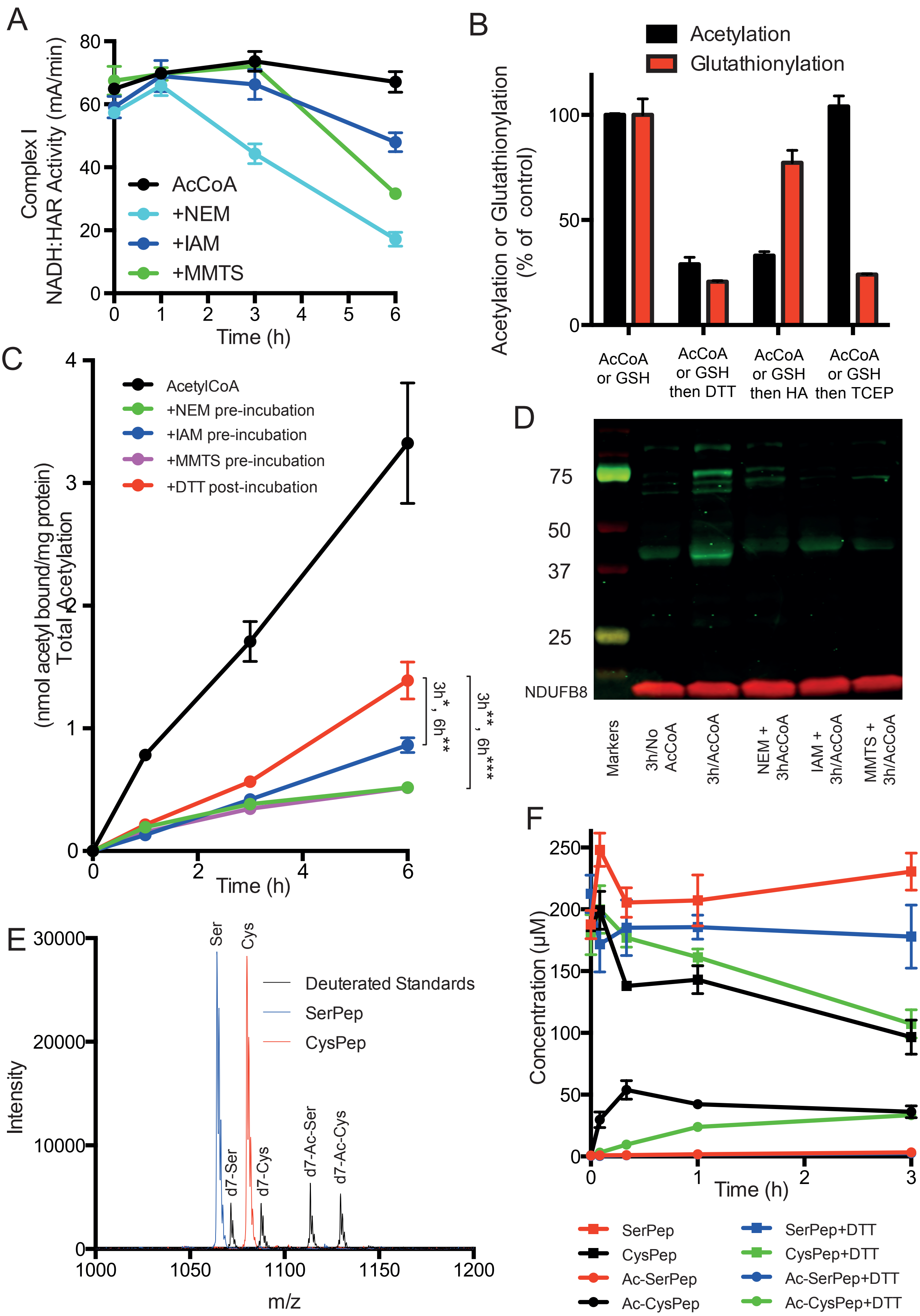

Figure S2. AcCoA acetylates lysine residues via a proximal acetylcysteine intermediate, Related to Figure 2.
A, sensitivity of complex I-dependent NADH:hexaammineruthenium (HAR) activity to thiol-blocking reagents. Bovine heart mitochondrial membrane fragments (5 mg/mL) were suspended in 20 μL of 50 mM NaH_2PO_4 (pH 7.8) supplemented with 25 mM NEM, IAM or MMTS. After incubation at 37˚C complex I-dependent NADH:HAR activity was measured. B, HA and TCEP selectively reduce thioesters and disulphides, respectively. Bovine heart mitochondrial membranes (5 mg/mL) were incubated with 2 mM ^{14}C-AcCoA or 5 mM ^{3}H-GSSG at 37˚C for up to 3 h. At the end of 3 h the reaction was quenched by addition of 1 mL of buffer containing 20 mM DTT, 20 mM TCEP or 50 mM HA. The sample was incubated for a further 30 min at 37˚C. The data are a percentage of the control mean ± SEM of at least 3 independent experiments. For ^{14}C-AcCoA the control mean was 1759 ± 12 nmol

^{14}C-Ac bound/mg protein. For ^{3}H-GSSG the control mean was 5174 ± 398 nmol ^{3}H-GS bound/mg protein. C, NEM, IAM and MMTS pre-incubation is significantly more effective at decreasing acetylation than post-incubation with a large excess of DTT. Bovine heart mitochondrial membranes (5 mg/mL) were incubated with 2 mM ^{14}C-AcCoA at 37˚C for up to 6 h. Some reactions were preincubated for 15 min at 37˚C with 25 mM NEM, IAM or MMTS. At the end of the incubation the reaction was quenched by addition of 1 mL of buffer. For DTT post-incubation this contained 20 mM DTT and the quenched sample was incubated for a further 30 min at 37˚C. The data are the mean ± SEM of at least 3 independent experiments. **, $p < 0.01$; ***, $p < 0.001$. D, *N*-acetylation is sensitive to thiol-blocking reagents. Mitochondrial membranes were incubated with 2 mM AcCoA and either 25 mM NEM, IAM or MMTS at 37˚C for 3 h. After reducing SDS-PAGE acetyllysine (green) and NDUFB8 (red) were visualised. a proximal thiol facilitates lysine acetylation. E, MALDI of synthetic peptides. Two synthetic peptides were designed, butyryl-RYAKGCASR-NH_2 (red; CysPep) and butyryl-RYAKGSASR-NH_2 (blue; SerPep). Four additional deuterated synthetic peptides (black) were made as standards, d7-butyryl-RYAKGCASR-NH_2 (d7-CysPep), d7-butyryl-RYAKGSASR-NH_2 (d7-SerPep), d7-butyryl-RYAK_{Ac}GCASR-NH_2 (d7-AcCysPep) and d7-butyryl-RYAK_{Ac}GSASR-NH_2 (d7-AcSerPep). F, a cysteine residue facilitates acetylation of nearby lysine and serine residues. CysPep (200 µM) and SerPep (200 µM) were coincubated with 200 µM AcCoA. For some 20 mM DTT was added for 30 min after the reaction to differentiate acetylcysteine from acetyllysine or acetylserine. A mix of deuterated (d7) standards were then added to quantify acetylation (Ac-SerPep and Ac-CysPep) by MALDI-TOF. Acetylation was calculated relative to the acetyllysine containing d7-AcCysPep and d7-AcSerPep standards. The roughly equimolar loss of CysPep and gain of DTT-sensitive AcCysPep at 5 min suggests acetylcysteine containing AcCysPep can be quantified using the d7-AcCysPep standard.

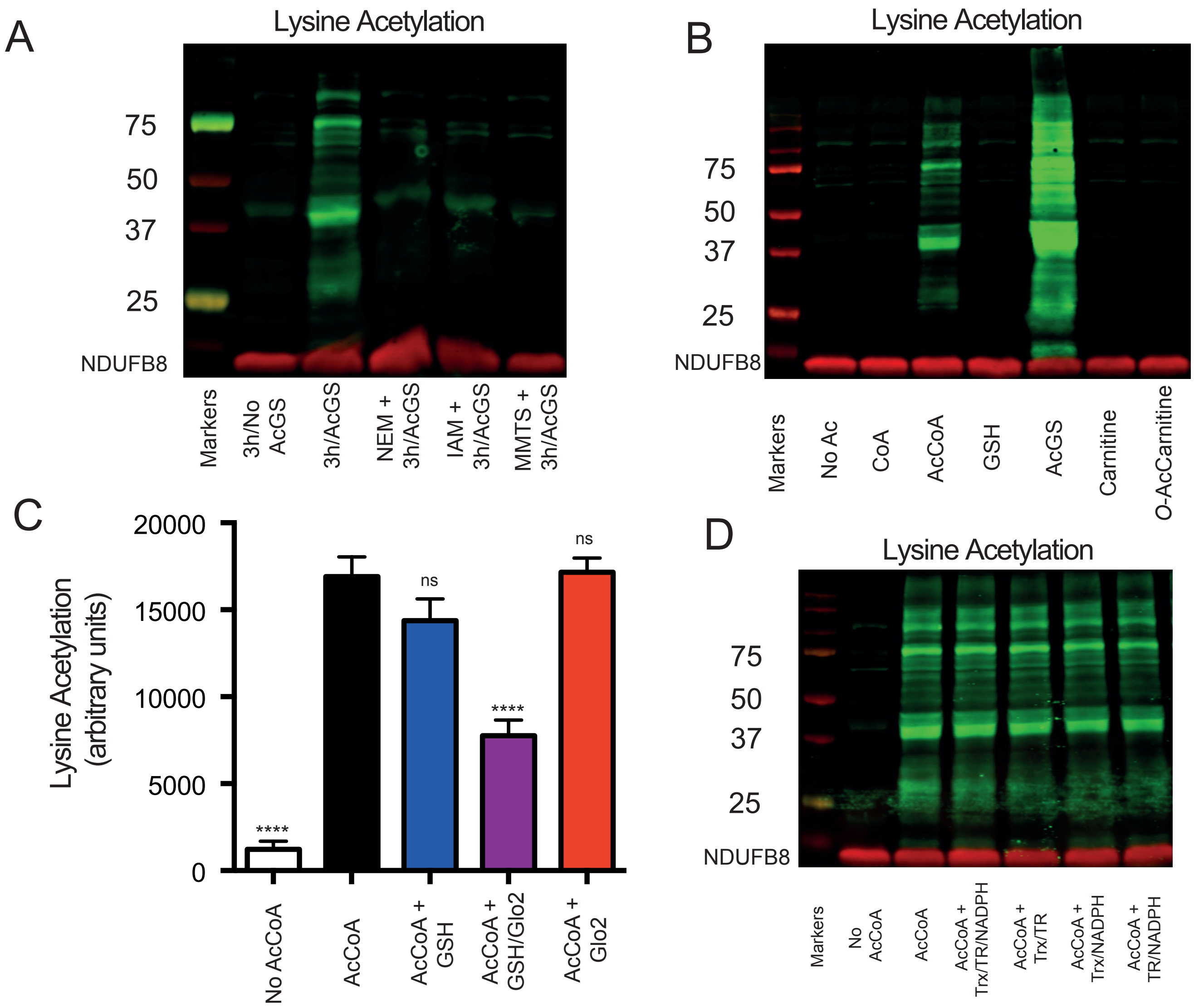

Figure S3. Acetylation is limited by glutathione and Glyoxalase II, Related to Figure 3.
A, *N*-acetylation is sensitive to thiol-blocking reagents. Mitochondrial membranes were incubated with 2 mM AcGS and either 25 mM NEM, IAM or MMTS at 37˚C for 3 h. After reducing SDS-PAGE acetyllysine (green) and NDUFB8 (red) were visualised. B, *O*-linked acetyl groups do not *N*-acetylate protein. Bovine heart mitochondrial membranes (5 mg/mL) were incubated with 2 mM CoA, AcCoA, GSH, AcGS, carnitine and *O*-acetylcarnitine at 37°C for 6 h. C, AcGS and AcCoA *N*-acetylate lysine residues and this is prevented by Glo2. Mitochondrial membranes were incubated with 2 mM AcCoA or 2 mM AcGS at 37˚C for 6 h. This was supplemented with GSH (10 mM) and/or Glo2 (1 µg/mL) during the 6h incubation as indicated. The data are the mean ± SEM of at least 3 independent experiments. ns, not significant; ****, $p < 0.0001$. D, thioredoxin reductase (TR) does not prevent *N*-acetylation of protein by AcCoA. Bovine heart mitochondrial membranes (5 mg/mL) were incubated with combinations of 2 mM AcCoA, 5 µM *E.coli* thioredoxin and 1 µg/mL *E.coli* TR, 1 mM NADPH for 6 h at 37˚C. After SDS-PAGE acetyllysine (green) and NDUFB8 (red) were visualised with fluorescent secondaries.

Table S1. Overlap of cysteine acylation and lysine acetylation, Related to Figure 4.
A list of mouse liver peptides containing both an acetyllysine (Rardin et al., 2013) and an acylcysteine (Gould et al., 2015). The acetyllysine dataset can be found in Table S4 (Rardin et al., 2013). The acylcysteine dataset can be found in Table S1 (Gould et al., 2015). The list includes peptides where the miscleaved acetyllysine containing peptide contains a cysteine residue that has a hydroxylamine-sensitive thioester modification. The sequences shown are longer than normal tryptic peptides because trypsin does not cleave at acetyllysine.
