# Supplementary material for: Non-enzymatic N-acetylation of Lysine Residues by AcetylCoA Often Occurs via a Proximal S-acetylated Thiol Intermediate Sensitive to Glyoxalase II
Source: Cell Rep. 2017 Feb 28;18(9):2105–12. doi: 10.1016/j.celrep.2017.02.018 (PMC6381604; doi:10.1016/j.celrep.2017.02.018)
Supplement: Document S2. Article plus Supplemental Information [file mmc3.pdf]

Cell Reports

Report

Non-enzymatic *N*-acetylation of Lysine Residues by AcetylCoA Often Occurs via a Proximal *S*-acetylated Thiol Intermediate Sensitive to Glyoxalase II

Graphical Abstract

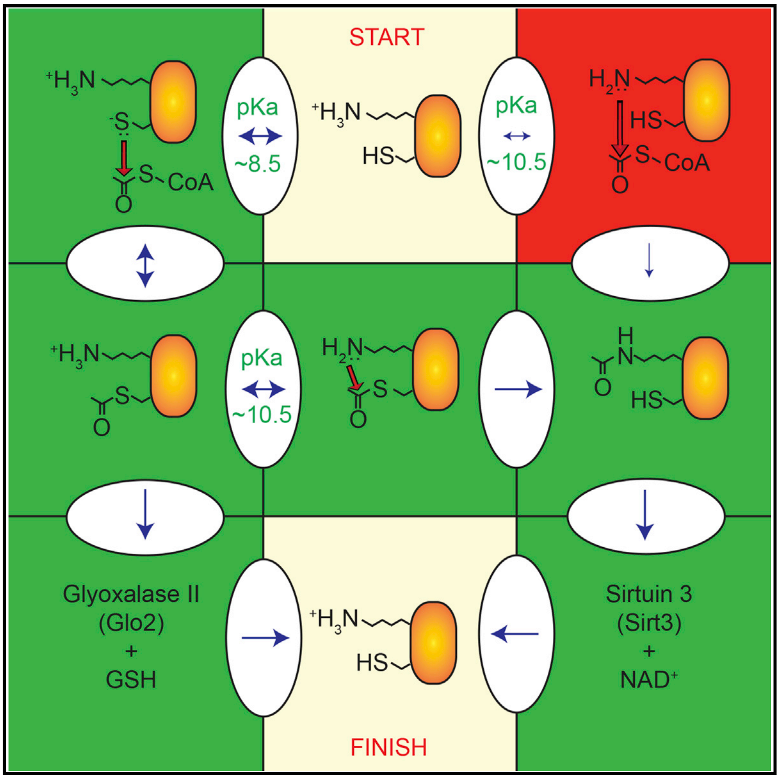

Highlights

- AcCoA and acetylglutathione reversibly acetylate protein cysteine residues
- Non-enzymatic lysine acetylation proceeds via a proximal *S*-acetylated thiol intermediate
- Glyoxalase II and glutathione limit lysine *N*-acetylation and *N*-succinylation
- These findings have implications for *N*-acetylation of lysines in regulation and pathology

Authors

Andrew M. James, Kurt Hoogewijs, Angela Logan, Andrew R. Hall, Shujing Ding, Ian M. Fearnley, Michael P. Murphy

Correspondence

aj@mrc-mbu.cam.ac.uk (A.M.J.), mpm@mrc-mbu.cam.ac.uk (M.P.M.)

In Brief

James et al. show that the non-enzymatic *N*-acetylation of lysine residues in mitochondrial proteins frequently occurs via a proximal *S*-acetylated thiol intermediate. Glutathione equilibrates with this intermediate, allowing the thioesterase glyoxalase II to limit protein lysine *N*-acetylation. These findings expand our understanding of how protein acetylation arises.

James et al., 2017, Cell Reports *18*, 2105–2112
February 28, 2017 © 2017 The Authors.
http://dx.doi.org/10.1016/j.celrep.2017.02.018

CellPress

Cell Reports
Report

OPEN ACCESS
CellPress

Non-enzymatic *N*-acetylation of Lysine Residues by AcetylCoA Often Occurs via a Proximal *S*-acetylated Thiol Intermediate Sensitive to Glyoxalase II

Andrew M. James,[1,*] Kurt Hoogewijs,[1,2,3] Angela Logan,[1] Andrew R. Hall,[1] Shujing Ding,[1] Ian M. Fearnley,[1] and Michael P. Murphy[1,4,*]
[1]Mitochondrial Biology Unit, Medical Research Council, Cambridge CB2 0XY, UK
[2]Laboratory of Molecular Biology, Medical Research Council, Cambridge CB2 0QH, UK
[3]The Wellcome Trust Centre for Mitochondrial Research, Institute for Cell and Molecular Biosciences, The Medical School, Newcastle University, Newcastle upon Tyne NE2 4HH, UK
[4]Lead Contact
*Correspondence: aj@mrc-mbu.cam.ac.uk (A.M.J.), mpm@mrc-mbu.cam.ac.uk (M.P.M.)
http://dx.doi.org/10.1016/j.celrep.2017.02.018

SUMMARY

Acetyl coenzyme A (AcCoA), a key intermediate in mitochondrial metabolism, *N*-acetylates lysine residues, disrupting and, in some cases, regulating protein function. The mitochondrial lysine deacetylase Sirtuin 3 (Sirt3) reverses this modification with benefits reported in diabetes, obesity, and aging. We show that non-enzymatic lysine *N*-acetylation by AcCoA is greatly enhanced by initial acetylation of a cysteine residue, followed by *SN*-transfer of the acetyl moiety to a nearby lysine on mitochondrial proteins and synthetic peptides. The frequent occurrence of an *S*-acetyl intermediate before lysine *N*-acetylation suggests that proximity to a thioester is a key determinant of lysine susceptibility to acetylation. The thioesterase glyoxalase II (Glo2) can limit protein *S*-acetylation, thereby preventing subsequent lysine *N*-acetylation. This suggests that the hitherto obscure role of Glo2 in mitochondria is to act upstream of Sirt3 in minimizing protein *N*-acetylation, thus limiting protein dysfunction when AcCoA accumulates.

INTRODUCTION

Acetyl coenzyme A (AcCoA) is central to mitochondrial metabolism, providing acetyl groups to the citric acid cycle from the oxidation of carbohydrate and fat (Pietrocola et al., 2015). One way that AcCoA affects mitochondria is through *N*-acetylation of the ε-amino of lysine residues on mitochondrial proteins. The significance of *N*-acetylation is implied by the existence of a mitochondrial deacetylase, sirtuin 3 (Sirt3), which uses NAD^+ to remove acetyl groups from lysine residues, and by the observation that Sirt3 is important in the pathology of a range of degenerative diseases, including cancer, aging, and diabetes (McDonnell et al., 2015).

Although the enzymatic basis of the deacetylation of *N*-acetylated lysine residues by Sirt3 is established, the mechanism by which mitochondrial AcCoA acetylates lysine residues is less certain. Although a mitochondrial *N*-acetyltransferase (Gcn5L1) has been proposed (Scott et al., 2012), more interesting in the context of degenerative disease is the observation that mitochondrial protein is non-enzymatically *N*-acetylated on lysine residues by AcCoA (Wagner and Payne, 2013; Wagner and Hirschey, 2014; Weinert et al., 2015; Davies et al., 2016). The mitochondrial concentration of coenzyme A (CoA) in vivo (~1 mM) is usually higher than AcCoA (~100–500 μM) (Garland et al., 1965), but this shifts in the presence of fatty acids with AcCoA rising (~1 mM) and CoA falling (~100–300 μM). Consequently, both the mitochondrial concentration of AcCoA and the AcCoA/CoA ratio alter markedly upon changes to nutrition or exercise. Whether protein lysine acetylation is a damaging consequence of relying on AcCoA for metabolism or a regulatory pathway to respond to changes in AcCoA, the AcCoA/CoA ratio or NAD^+ is unclear. Even so, Sirt3 plays a major pathophysiological role in reversing the damaging lysine *N*-acetylation of mitochondrial proteins that arises from exposure to excess AcCoA.

The mechanism proposed for non-enzymatic lysine acetylation was a nucleophilic attack by the side-chain amine on the acetyl carbonyl (Wagner and Payne, 2013). This direct reaction will be slow in the mitochondrial matrix (pH ~7.8), because the pK_a of a free lysine is ~10.5. Although this rate can be enhanced by stabilization of the amine, protein cysteine residues are on average better nucleophiles (pK_a ~8.5) (Thapa et al., 2014). Because cysteine-containing peptides can be directly *S*-acetylated by AcCoA in vitro (Bizzozero et al., 2001), we postulated that *S*-acetylation of protein cysteine thiols would be far higher than *N*-acetylation of lysine amines. Here we show that *S*-acetylation is a frequent modification and that non-enzymatic *N*-acetylation of lysine residues by AcCoA occurs predominantly via a proximal *S*-acetylated thiol intermediate. We also show that glutathione (GSH) and glyoxalase II (Glo2), also known as hydroxyacyl glutathione hydrolase (HAGH), together can limit *S*-acetylation and consequently *N*-acetylation.

This is an open access article under the CC BY license (http://creativecommons.org/licenses/by/4.0/).

OPEN
ACCESS
CellPress

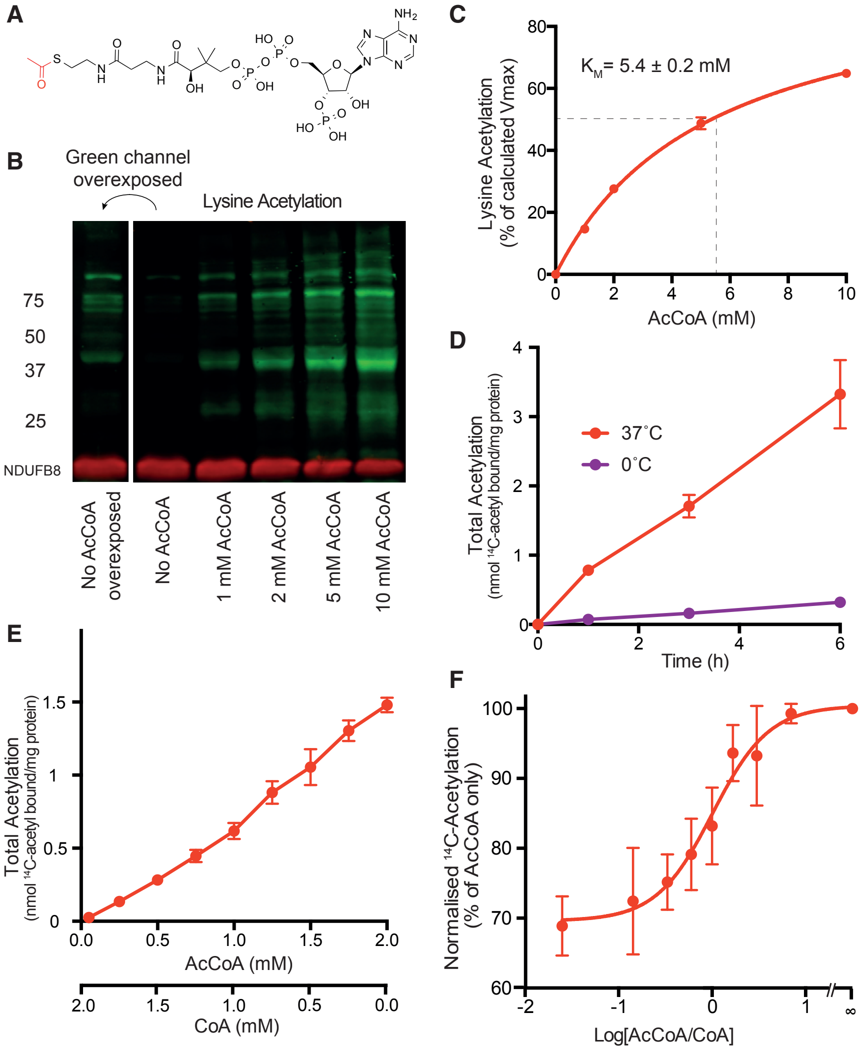

Figure 1. AcCoA Non-enzymatically Acetylates Mitochondrial Protein

(A) Structure of AcCoA.

(B and C) Lysine acetylation by AcCoA is concentration dependent, has a high apparent K_M, and resembles in vivo acetylation. (B) Mitochondrial membranes were incubated with AcCoA at 37°C for 6 hr, followed by reducing SDS-PAGE. The green channel without AcCoA was overexposed for comparison. (C) After SDS-PAGE, acetyllysine (green) and NDUFB8 (red) were visualized (n = 2 ± range). (D) Total acetylation is temperature sensitive. Mitochondrial membranes were incubated with 2 mM ^{14}C-AcCoA on ice or at 37°C.

(E) Total acetylation is concentration dependent. Mitochondrial membranes were incubated with 50 μM ^{14}C-AcCoA and 0–2 mM AcCoA at 37°C. The reaction contained sufficient CoA to keep AcCoA+CoA at 2 mM. Bound acetyl was calculated using the combined specific activity of ^{14}C-AcCoA and AcCoA.

(F) Acetylation is influenced by CoA. Incubations as for (E) but recalculated as ^{14}C-acetyl bound (dpm) irrespective of specific activity. This was normalized to 2 mM AcCoA (∞).

All data are the mean ± SEM of at least three experiments unless otherwise stated. See also Figure S1.

RESULTS

Mitochondrial Proteins Are Acetylated by AcCoA

It has been shown using an anti-acetyllysine antibody that mitochondrial proteins can be non-enzymatically *N*-acetylated on lysine residues by AcCoA (Wagner and Payne, 2013). To explore this further, we incubated bovine heart mitochondrial membrane fragments that lack metabolites or matrix proteins with AcCoA. Lysine acetylation was concentration dependent (Figure 1B) in the physiological range for AcCoA with an apparent K_M of 5.4 mM (Figure 1C). The anti-acetyllysine antibody recognizes acetyllysine modifications (Figures S1A and S1B), and the pattern of lysine acetylation observed in this in vitro system resembles that of in vivo lysine acetylation (Figure 1B).

The mechanism proposed for lysine *N*-acetylation is via nucleophilic attack on AcCoA by the side-chain amine of a lysine residue (Wagner and Payne, 2013). However, the pK_a of a lysine amine is ~10.5, making the more nucleophilic $-NH_2$ form a minor species in vivo without stabilization. In contrast, cysteine residues are better nucleophiles at physiological pH, with significant amounts of the thiolate available for reaction (pK_a ~8.5). Consequently, a major proportion of protein acetylation may be invisible to the anti-acetyllysine antibodies commonly used. To investigate total acetylation of mitochondrial protein by AcCoA, we incubated mitochondrial membranes with ^{14}C-AcCoA. As expected, membrane-associated ^{14}C increased with time and temperature (Figure 1D), consistent with a reaction between AcCoA and protein. The in vivo concentration of AcCoA plus CoA is likely to be stable ~1–2 mM (Garland et al., 1965). To reflect this, we incubated mitochondrial membranes with 50 μM ^{14}C-AcCoA supplemented with varying ratios of AcCoA and CoA to a total concentration of 2 mM AcCoA+CoA. The rate of protein acetylation was a function of the AcCoA concentration (Figure 1E) and the AcCoA/CoA ratio (Figure 1F) in the physiological range for AcCoA.

Thus, acetylation of mitochondrial proteins occurs in vitro in a time-, temperature-, and concentration-dependent manner without matrix proteins, other water-soluble metabolites, or cofactors. It has a high apparent K_M, and the pattern of proteins modified resembles in vivo acetylation.

Mitochondrial Proteins Are *S*-acetylated by AcCoA

To test whether AcCoA modified cysteine residues we included the thiol-alkylating reagent, *N*-ethyl maleimide (NEM; 25 mM). NEM reacts irreversibly with thiols and should block the transfer of the acetyl group of AcCoA to cysteines. Although NEM is

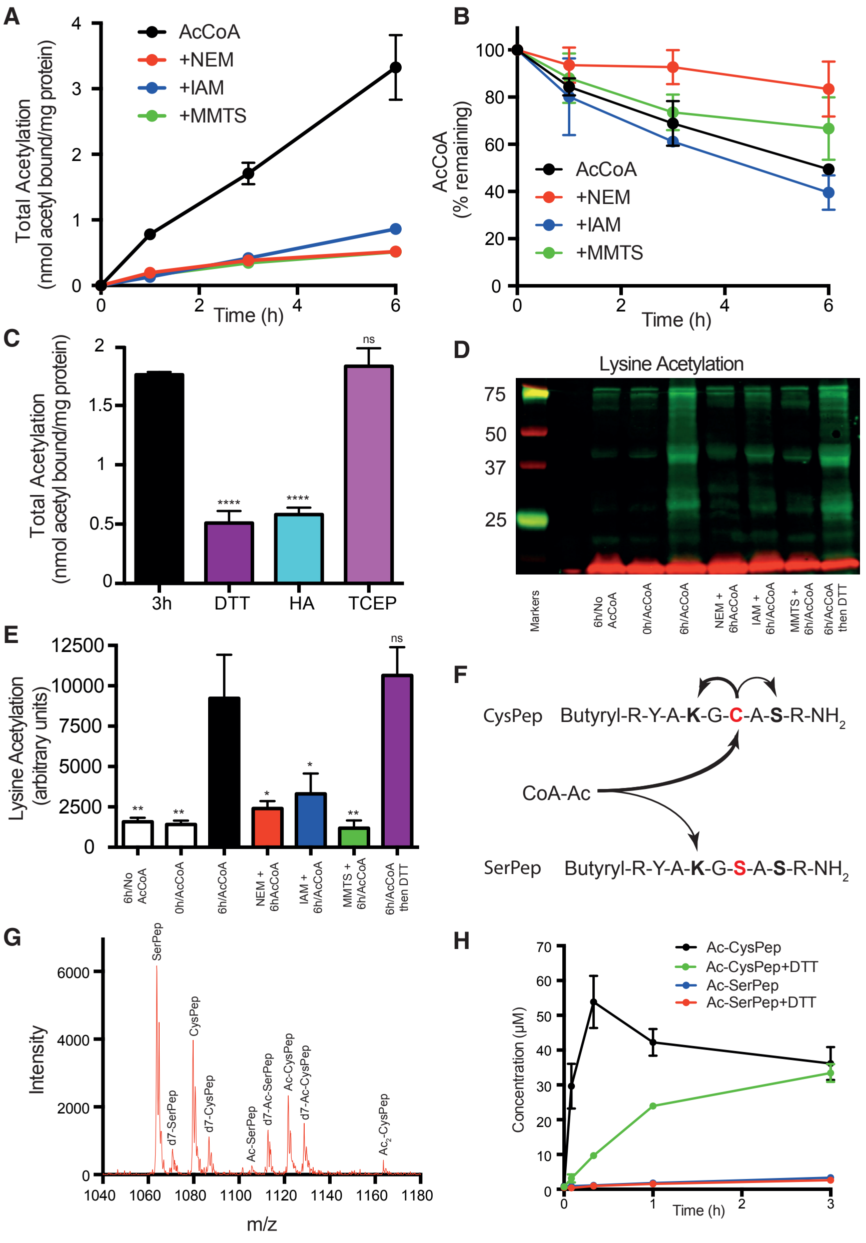

Figure 2. AcCoA Acetylates Lysine Residues via a Proximal Acetylcysteine Intermediate

(A) Total acetylation is sensitive to thiol-blocking reagents. Mitochondrial membranes were incubated with 2 mM ^{14}C-AcCoA and 25 mM NEM, IAM, or MMTS at 37°C for up to 6 hr.

(B) Alkylating reagents do not break down AcCoA. Mitochondrial membranes were incubated with 2 mM AcCoA and 25 mM NEM, IAM, or MMTS at 37°C for up to 6 hr. AcCoA was measured by liquid chromatography-tandem mass spectrometry (LC-MS/MS) (n = 2 ± range).

(C) Total acetylation is sensitive to thioester-cleaving reagents. Mitochondrial membranes were incubated with 2 mM ^{14}C-AcCoA at 37°C for 3 hr. Afterward, 20 mM DTT, HA, or TCEP was added for 30 min at 37°C.

(D and E) *N*-acetylation is sensitive to thiol-blocking reagents. (D) Mitochondrial membranes were incubated with 2 mM AcCoA and 25 mM NEM, IAM, or MMTS at 37°C for 6 hr. For others, 20 mM DTT was added for 30 min after the reaction. After reducing SDS-PAGE, acetyllysine (green) and NDUFB8 (red) were visualized. (E) The extent of lysine acetylation was quantified (n = 3 ± SD).

(F–H) Proximal thiol facilitates lysine acetylation as is shown in (F). A cysteine-containing peptide (CysPep; 200 μM) and a serine-containing peptide (SerPep; 200 μM) were coincubated with 200 μM AcCoA. To differentiate *S*-acetylcysteine from *N*-acetyllysine and *O*-acetylserine, 20 mM DTT was added for 30 min after the reaction. A mix of deuterated (d7) standards was then added to quantify acetylation (Ac-SerPep and Ac-CysPep) by MALDI-TOF. The spectrum in (G) is after 3 hr with DTT added. (H) The extent of acetylation was quantified (n = 3 ± SD).

All data are the mean ± SEM of at least three experiments unless otherwise stated. NS, not significant; *$p < 0.05$; **$p < 0.01$; ****$p < 0.0001$. See also Figures S1 and S2.

selective for thiols, over long periods at pH 8, it also alkylates amines (Figures S1C and S1D) and can disrupt secondary structure (Figure S2A). Thus, we used two further thiol-blocking reagents, iodoacetamide (IAM; 25 mM) and methylmethanethiosulfonate (MMTS; 25 mM) that react more selectively with cysteine residues (Figures S1E and S1F). After 3 hr, 85%, 79%, and 83% of acetylation was prevented by NEM, IAM, and MMTS, respectively, suggesting substantial acetylation of cysteines by ^{14}C-AcCoA (Figure 2A). To ensure NEM, IAM, and MMTS were not degrading AcCoA, we measured the change in AcCoA concentration by electrospray ionization (ESI) mass spectrometry (MS) (Figure 2B). After 3 hr, 70% of the AcCoA remained, and NEM, IAM, and MMTS did not accelerate AcCoA degradation. Thus, the lack of acetylation with NEM, IAM, and MMTS is not because exposure to AcCoA is diminished.

Consistent with acetylation of cysteine thiols, 71% of bound ^{14}C could be removed by excess DTT (20 mM for 30 min), a broad specificity thiol reductant, after incubation with ^{14}C-AcCoA (Figure 2C). Acetylation was reversed to a similar degree (67%) by hydroxylamine (HA; 50 mM for 30 min), a nucleophile with specificity for thioesters (Figure S2B), after the incubation with ^{14}C-AcCoA (Figure 2C). Acetylation was insensitive to tris(2-carboxyethyl) phosphine (TCEP) (Figure 2C), a disulfide-reducing agent unreactive with thioesters (Figure S2B).

Acetyl Moieties Migrate from Cysteine to Lysine Residues

Removing acetyl groups with DTT post-incubation was less effective at decreasing protein acetylation than blocking protein

OPEN
ACCESS
CellPress

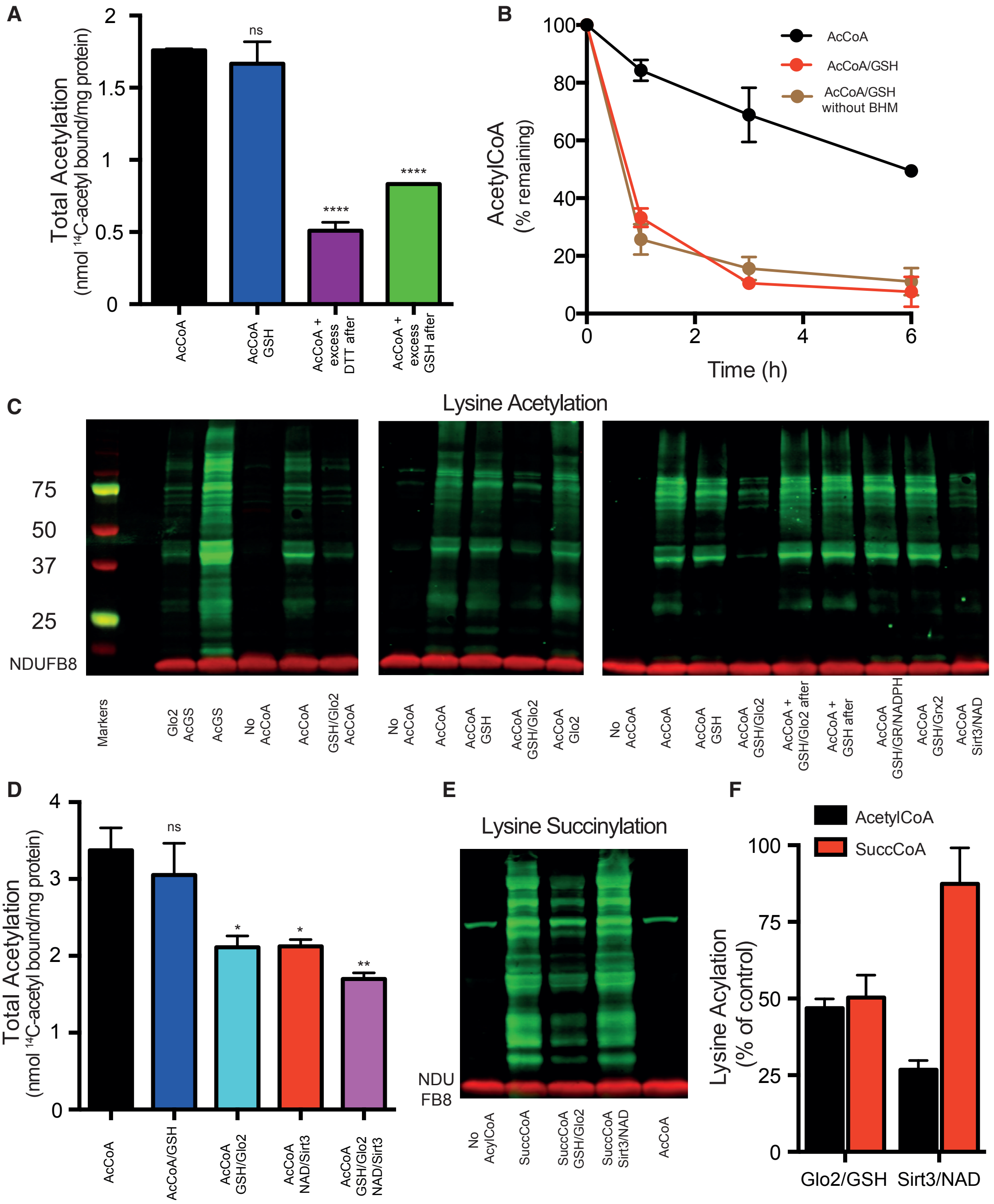

Figure 3. GSH and Glo2 Limit Acetylation

(A) Acetylation is insensitive to the presence of GSH. Mitochondrial membranes were incubated with 2 mM ^{14}C-AcCoA with or without 10 mM GSH at 37°C for 3 hr. At that point, an excess of DTT or GSH was added to some incubations for 30 min at 37°C.

(B) GSH breaks down AcCoA. AcCoA (2 mM) was incubated with or without 10 mM GSH and with or without mitochondrial membranes at 37°C for up to 6 hr. AcCoA was measured by LC-MS/MS. Data are the mean ± range of two experiments.

(C) *S*-acetylglutathione and AcCoA *N*-acetylate lysine residues are shown, and this is prevented by Glo2. Mitochondrial membranes were incubated with 2 mM AcCoA or 2 mM *S*-acetylglutathione at 37°C for 6 hr. This was supplemented with GSH (10 mM), NAD$^+$ (1 mM), NADPH (1 mM), Glo2 (1 μg/mL), GR (0.1 μg/mL), Grx2 (1 μg/mL), or Sirt3 (10 μg/mL) during the 6 hr incubation. Excess GSH was then added for 30 min as indicated. After reducing SDS-PAGE, acetyllysine (green) and NDUFB8 (red) were visualized.

(D) Acetylation by AcCoA is limited by Glo2 and Sirt3. Mitochondrial membranes were incubated with 2 mM ^{14}C-AcCoA at 37°C for 6 hr. GSH (10 mM), Glo2 (1 μg/mL), NAD$^+$ (1 mM), and Sirt3 (10 μg/mL) were added as indicated.

(legend continued on next page)

thiols with NEM, IAM, or MMTS preincubation, and the relative ineffectiveness of DTT increased with time (Figure S2C). Intramolecular *SN*-transfer reactions are often favorable and are used by enzymes such as acetyltransferases (Yan et al., 2002), as well as during native chemical ligation of peptides (Thapa et al., 2014). An increasing difference with time between preincubation with NEM, IAM, and MMTS and post-incubation with DTT (Figure S2C) could arise if AcCoA first *S*-acetylates a cysteine thiol through reversible thioester exchange and the acetyl moiety subsequently migrates in an irreversible intramolecular *SN*-transfer reaction to a nearby lysine. Consistent with this, preincubation of membranes with NEM, IAM, and MMTS all decreased lysine *N*-acetylation by western blot (Figures 2D, 2E, and S2D). In contrast to ^{14}C-AcCoA (Figure 2C), post-incubation with DTT failed to remove the *N*-acetylation signal resulting from AcCoA.

To show this intramolecular *SN*-transfer reaction is a general proximity-based process that can occur on amino acid side chains without a favorable secondary structure that might bind AcCoA or stabilize nucleophiles, two peptides were synthesized that contained either a cysteine (CysPep) or a serine (SerPep) near a lysine residue (Figure 2F). Four deuterated standards were also made to facilitate quantification by MS (Figures S2E). When 200 μM of both peptides were simultaneously coincubated with 200 μM AcCoA in a competition assay, DTT-sensitive acetylation of CysPep rapidly increased, peaking at 20 min (Figure 2H). The initial rate of total acetylation of CysPep was ~300-fold greater than that of SerPep, confirming the favorability of *S*-acetylation relative to direct *N*-acetylation. DTT-insensitive acetylation of CysPep (m/z = 1,122) continued to increase and was 23-fold higher (n = 3, $p < 0.0001$) than that of SerPep (m/z = 1,106) after 1 hr. At 3 hr, an additional DTT-insensitive peak (m/z = 1,164) appeared, consistent with CysPep acetylated on both lysine and serine (Figure 2G), and these modifications were confirmed by MALDI-TOF-TOF MS. Thus, initial reversible *S*-acetylation of a cysteine thiol via rapid thioester equilibration can facilitate subsequent *SN*-transfer of the acetyl moiety to a nearby lysine or serine residue. The rate of serine residue acetylation (pK_a ~13) is expected to be slower than for lysine.

GSH Alone Does Not Prevent *S*-acetylation

High concentrations of the free thiol GSH could limit acetylation of protein thiols in vivo. Thus, we tested whether protein thiols could be acetylated in the presence of physiological concentrations of GSH (10 mM) and AcCoA (2 mM) and a physiological GSH-to-AcCoA ratio of 5 (Figure 2C). Surprisingly, 10 mM GSH during the 3 hr incubation with AcCoA only prevented 5% of the acetylation (Figure 3A). In contrast, an unphysiological 2,000-fold excess of GSH to protein thiols, added after the reaction of protein with AcCoA, removed 53% of the protein-bound acetyl moieties (Figure 3A), a level approaching DTT and HA (Figure 2C). Thus, *S*-acetylation of cysteine thiols still occurs with physiological concentrations of AcCoA and GSH but is reversible with excess GSH.

Glo2 and GSH Limit Mitochondrial Protein Acetylation

Despite suggesting limited direct interaction between GSH and AcCoA, GSH caused a rapid decline in AcCoA (Figure 3B) and the appearance of a mass consistent with acetylglutathione (m/z = 348). This reaction did not require the presence of protein (Figure 3B) and is consistent with thioester equilibration between AcCoA and GSH. One explanation for why GSH did not prevent protein acetylation, despite depleting AcCoA, is that *S*-acetylglutathione (AcGS) also acetylates proteins. Incubation of mitochondrial membranes with 2 mM *S*-acetylglutathione (Figure 3C) led to *N*-acetylation of lysine residues increasing 44-fold. Although the pattern of protein acetylation was similar to that caused by AcCoA alone, the extent of lysine modification was 3.1 ± 0.2-fold higher. Acetylation by *S*-acetylglutathione was also sensitive to NEM, IAM, and MMTS (Figure S3A). There was no acetylation by *O*-linked acetylcarnitine (Figure S3B).

S-acetylglutathione is a good substrate for Glo2, an enzyme that hydrolyses a range of thioesters conjugated to GSH (Talesa et al., 1989). Glo2 catalyzes the second stage of the two-step detoxification pathway for glyoxals that form during glycolysis in the cytoplasm. Glyoxalase I (Glo1) conjugates these dicarbonyls to GSH via a thioester bond, and Glo2 subsequently recognizes the GSH moiety and cleaves this thioester. Mitochondria contain Glo2, but not Glo1, suggesting that mitochondrial Glo2 has an alternate role (Rabbani et al., 2014). When human Glo2 (20 ng, 0.5 mU) was added to an incubation of *S*-acetylglutathione and mitochondrial membranes, *N*-acetylation decreased by 68% ± 9% (Figure 3C). Next, we tested whether *N*-acetylation by 2 mM AcCoA was also prevented by Glo2 (Figures 3C and S3B). When GSH was combined with Glo2, *N*-acetylation decreased by 54% ± 4% (n = 7, $p < 0.0001$). However, neither GSH nor Glo2 alone significantly decreased *N*-acetylation by AcCoA (Figures 3C and S3C).

Mitochondria contain mechanisms to recycle disulfides that might also act on *S*-acetylglutathione or acetylated cysteine residues. GSH reductase (GR) reduces glutathione disulfide (GSSG) using NADPH, and the addition of yeast GR (2 ng, 0.44 mU), 1 mM NAPDH, and 10 mM GSH did not decrease protein *N*-acetylation (Figure 3C). Glutaredoxin 2 (Grx2) reduces protein-GSH mixed disulfides in the presence of GSH, and the addition of human Grx2 (20 ng, 0.4 mU) with 10 mM GSH also failed to decrease protein *N*-acetylation (Figure 3C). Protein disulfides can be reduced by thioredoxin reductase (TR) and thioredoxin (Trx), but the addition of TR (20 ng, 0.4 mU), Trx (5 μM), and 1 mM NADPH had no effect on protein acetylation (Figure S3D). Mitochondria contain Sirt3, which can remove acetyl groups from lysine residues. As expected, human recombinant Sirt3 (200 ng, 0.0004 mU) with 1 mM NAD^+ reversed protein *N*-acetylation by AcCoA by 70% ± 4% (Figure 3C).

Next, we measured whether total protein acetylation by ^{14}C-AcCoA was sensitive to GSH/Glo2 or NAD^+/Sirt3 alone or in combination. Incubation with 2 mM AcCoA for 6 hr led to protein acetylation, and this was decreased 37% ± 2% or

(E and F) *N*-succinylation by succinylCoA (SuccCoA) is limited by Glo2, but not Sirt3. Mitochondrial membranes were incubated with 2 mM SuccCoA or AcCoA at 37°C for 6 hr. This was supplemented with GSH (10 mM), NAD^+ (1 mM), Glo2 (1 μg/mL), or Sirt3 (10 μg/mL) during the 6 hr incubation as indicated. (E) After SDS-PAGE, succinyllysine (green) and NDUFB8 (red) were visualized. (F) The extent of lysine acetylation was quantified (n = 3 ± SD).
All data are the mean ± SEM of at least three experiments unless otherwise stated. NS, not significant; *$p < 0.05$; **$p < 0.01$; ****$p < 0.0001$. See also Figure S3.

OPEN
ACCESS
CellPress

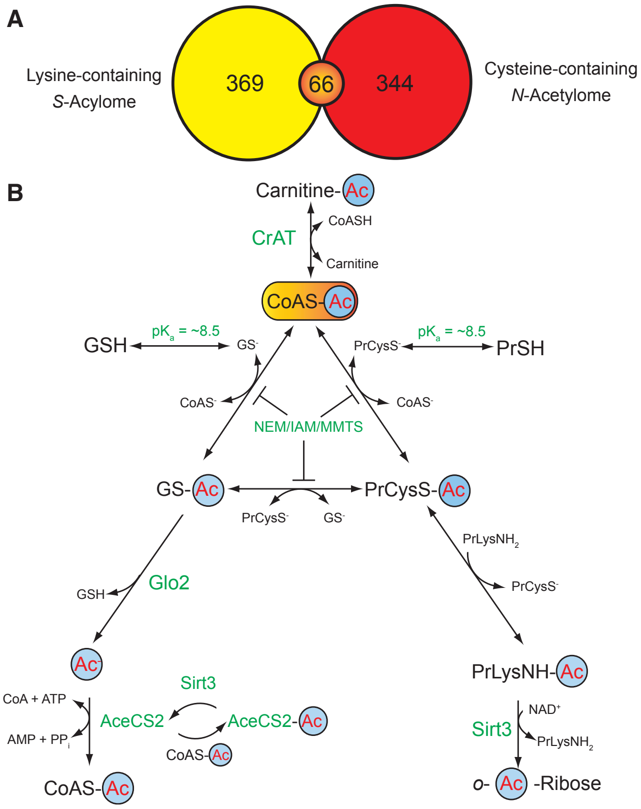

Figure 4. Model of Thiol Involvement in Non-enzymatic Protein Acetylation

(A) Coexistence of *N*-acetyllysine and *S*-acylcysteine. Overlap (orange) between lysine-containing peptides in the mouse liver *S*-acylome (yellow) (Gould et al., 2015) and cysteine-containing peptides (red) in the mouse liver mitochondrial *N*-acetylome (blue) (Rardin et al., 2013).

(B) Model of non-enzymatic acetylation. The steady-state concentration of reactive AcCoA is buffered lower by carnitine and carnitine acetyltransferase (CrAT). The pK_a and relative concentrations of GSH and protein thiols (PrCysSH) determine the rate at which AcCoA reacts with their thiolate forms, GS^- and $PrCysS^-$. NEM, IAM, and MMTS limit reversible thioester exchange by blocking thiols. Glo2 shifts the equilibrium away from cysteine-bound acetyl groups (PrCysSAc) by hydrolyzing *S*-acetylglutathione to acetate (Ac^-). PrCysSAc not removed by GSH can irreversibly transfer to a nearby lysine ($PrLysNH_2$), where Sirt3 can degrade it. AcCoA is regenerated by AcCoA synthetase (AceCS2), and this is regulated by *N*-acetylation and Sirt3.
See also Figure S4 and Table S1.

37% ± 3% by the presence of GSH/Glo2 or NAD^+/Sirt3, respectively (Figure 3D). Glo2 and Sirt3 in combination decreased acetylation by 53% ± 3%.

Glo2 Can Limit Acylation by Other AcCoA Species

Although AcCoA is the most common form of activated CoA in vivo, it is not the only AcCoA present. CoA is used extensively as a cofactor in metabolism, and many other moieties are conjugated to its thiol, e.g., succinylCoA in the tricarboxylic acid cycle. One potential advantage of GSH/Glo2 over NAD^+/Sirt3 is that Glo2 is selective for GSH, but not the acyl moiety conjugated to its thiol (Talesa et al., 1989; Vander Jagt, 1993). To test whether Glo2 could diminish succinylation, we incubated mitochondrial membranes with 2 mM succinylCoA. GSH/Glo2, but not NAD^+/Sirt3, could decrease lysine succinylation (Figures 3E and 3F). Thus, GSH and Glo2 provide a broad specificity mechanism for removing AcCoA-dependent modifications on cysteine residues and consequently limiting their accumulation on lysine residues.

DISCUSSION

Although reactions between AcCoA and cysteine thiols have been noted previously (Bizzozero et al., 2001), almost all acetylation literature focuses on *N*-acetylation of lysine residues by acetyltransferase enzymes (McDonnell et al., 2015). It has been recognized that *N*-acetylation can also occur non-enzymatically (Wagner and Payne, 2013; Wagner and Hirschey, 2014; Weinert et al., 2015; Davies et al., 2016). Our results show that non-enzymatic *N*-acetylation frequently occurs indirectly via the reversible *S*-acetylation of a nearby thiol. This thioester exchange, followed by an intramolecular *SN*-transfer reaction to a proximal amine, underlies the native chemical ligation of peptides (Thapa et al., 2014), autoacetylation of tau protein (Cohen et al., 2013), and reactions in the active sites of enzymes such as acetyltransferases (Yan et al., 2002; Cohen et al., 2013). Here we show that this intramolecular *SN*-transfer reaction is likely to be widespread, because it proceeds rapidly on a peptide lacking secondary structure (Figures 2G and 2H). Although secondary structure is not a requirement (Figure 2H) and the apparent K_M of the reaction is high (Figure 1C), we would expect enhancement of lysine acetylation, in which the secondary structure stabilizes a nucleophile or leads to AcCoA associating with a protein. AcCoA shares an ADP-based backbone with ATP, NAD(P)H, and flavin adenine dinucleotide (FADH), and Rossmann-fold binding sites for these cofactors are prevalent throughout the cell.

Our use of HA to selectively cleave the thioester bond was based on its widespread use to study *S*-acylation-dependent membrane anchoring by *S*-acyltransferases and palmitoylCoA (Roth et al., 2006). Consequently, some of the 627 *S*-acylcysteine residues identified by MS in a mouse liver *S*-acylation dataset could have been *S*-acetylated by AcCoA (Gould et al., 2015). Merging this dataset with the 344 acetyllysines on peptides that also contain a cysteine residue in a mouse liver mitochondrial acetylome (Rardin et al., 2013) shows 66 acetyllysine modifications on peptides, which also contained a *S*-acylated cysteine residue (Figure 4A; Table S1). Thus, a number of acetylcysteine and acetyllysine residues coexist in vivo and may be worthy of further study.

Enrichment with anti-acetyllysine antibodies and improvements in MS have identified an increasing number of lysine acetylation sites (Rardin et al., 2013). However, purification of acetylated from unacetylated peptides limits understanding of stoichiometry and physiological importance (Weinert et al., 2015). Bovine heart mitochondrial membranes contain 53 nmol of exposed thiols per milligram of protein (Requejo et al., 2010), and 0.78% of exposed cysteine thiols were acetylated every hour by 2 mM AcCoA (see Supplemental Information). This corresponds to 0.13% of exposed lysine residues *N*-acetylated each hour. Our in vitro concentration of exposed thiols is ~265 μM, which is ~100-fold lower than in vivo; consequently, the rate of the reaction between AcCoA and protein could be

much higher. However, basal *N*-acetylation of the mitochondrial membrane preparation, a function of acetylation and deacetylation, is 17% of that after 6 hr with 2 mM AcCoA (Figure 2F). This is equivalent to 0.13% of exposed lysine residues, a value consistent with the median *N*-acetylation stoichiometry of liver mitochondrial proteins (0.11%) observed by MS (Weinert et al., 2015). Thus, it is likely, at least in mitochondria, that most acetylome arises from low-level non-enzymatic acetylation.

The low stoichiometry of residue modification suggests this process is analogous to oxidative stress, in which reactive oxygen species (ROS) non-enzymatically modify protein, DNA, and lipid. One striking difference is that AcCoA is central to metabolism, so it cannot be directly degraded. This limits protective strategies to preventing excess AcCoA and removal of acetyl groups from proteins when they form. In addition to Sirt3, it has been shown that carnitine acetyltransferase (CrAT) buffers the AcCoA concentration by storing excess AcCoA as unreactive *O*-acetylcarnitine (Figure S3B), thus limiting the thioester concentration and consequently cysteine and lysine acetylation (Davies et al., 2016). Here we identify another system capable of limiting CoA-derived modifications: the mitochondrial GSH and Glo2 system (Figure 4B). Cytosolic Glo2 and Glo1 detoxify glyoxals formed during glycolysis; however, an alternate function is required for mitochondrial Glo2, because the matrix lacks glycolysis and Glo1 (Rabbani et al., 2014). We suggest that a major function of matrix Glo2 is to degrade *S*-acetylglutathione and thereby shift the equilibrium away from cysteine *S*-acetylation, thus limiting acetylation of vital cysteine and lysine residues (Figure 4B). GSH at physiological concentrations is ineffective, because the product of the reaction is *S*-acetylglutathione, which could simply reacetylate protein, as thioester exchange is faster than non-enzymatic hydrolysis (Bracher et al., 2011). Glo2 is advantageous, because it increases the rate of hydrolysis of a range of acylglutathione species (Talesa et al., 1989) without affecting their corresponding AcCoAs.

Enzyme active sites have evolved over time to be efficient, and many have intermediate steps with substrate covalently linked to active site residues. We suspect protein modifications can result from unintentional chemistry on less evolved protein surfaces, where the second step releasing the modification is absent or the target is a proximal amino acid. The AcCoA concentration is ~1,000-fold greater than most ROS and AcCoA cannot be scavenged; together this represents both a problem for protein function and an opportunity for regulation. Perhaps these regulatory pathways arose because the enzymes required to switch them off, sirtuins, had already evolved to prevent non-specific damage to protein.

Although many of the low-occupancy, potentially deleterious acetylation sites identified by MS may arise from the *SN*-transfer described here, highly acetylated regulatory proteins may use a combination of features to achieve a robust desirable signal. Consequently, the relative contribution of proximal cysteine thiols within signaling pathways would need to be explored case by case with cysteine mutations. Furthermore, although this work provides a potential explanation for the expression of Glo2 in mitochondria, the physiological relevance of *S*-acylation is still unknown and will need to be determined in vivo by the use of knockout animals.

EXPERIMENTAL PROCEDURES

Materials

Recombinant human Glo2 (25 nmol/min/μg) and Sirt3 (2 pmol/min/μg) were from R&D Systems. Rabbit anti-acetyllysine (9441) was from Cell Signaling Technology. Rabbit anti-succinyllysine (PTM-401) was from PTM Biolabs. Bovine heart mitochondrial membrane fragments were prepared as described previously and stored at −80°C until used (Sharpley et al., 2006).

Radioactive Acetylation of Protein

Mitochondrial membrane fragments (5 mg/mL) were suspended in 20 μL of KP_i buffer (50 mM KH_2PO_4, 100 μM EDTA, 100 μM diethylenetriaminepentaacetic acid (DTPA) [pH 7.8]) supplemented with NEM (25 mM), IAM (25 mM), MMTS (25 mM), human Glo2 (1 μg/mL), human Sirt3 (10 μg/mL), NAD^+ (1 mM) or GSH (10 mM) as indicated. When NEM, IAM, and MMTS were used, they were preincubated with mitochondrial membrane fragments for 15 min at 37°C before the addition of AcCoA. The reaction was started with 50 μM ^{14}C-AcCoA and a 2 mM mixture of non-radioactive AcCoA and CoA before incubation for up to 6 hr at either 0°C or 37°C. The reaction was quenched by the addition of 980 μL of KP_i buffer. To this 1 mL volume, a large excess of DTT (20 mM), HA (50 mM), TCEP (20 mM), or GSH (20 mM) was sometimes added, followed by a further 30 min incubation at 37°C. Samples were spun at 16,000 × *g* for 10 min with the supernatant discarded and the pellet washed with a further 1 mL of 50 mM KP_i buffer. The supernatant was discarded, and the inside of the tube dried. The pellet was resuspended in 50 μL of 20% (v/v) Triton X-100. When ^{14}C-AcCoA was added after the incubation, ^{14}C counts in the pellet water space were ~5% of the total at 6 hr. EDTA and DTPA were omitted from the buffer for experiments with Glo2 (1 μg/mL) and Sirt3 (10 μg/mL). The specific activity of recombinant Sirt3 is much lower than recombinant Glo2, hence the addition of ten times as much protein.

Lysine Acetylation

Mitochondrial membrane fragments (5 mg/mL) were suspended in 20 μL of NaP_i buffer (50 mM NaH_2PO_4, 100 μM EDTA, 100 μM DTPA [pH 7.8]) supplemented with NEM (25 mM), IAM (25 mM), MMTS (25 mM), human Glo2 (1 μg/mL), human Sirt3 (10 μg/mL), human Grx2 (1 μg/mL), yeast GR (0.1 μg/mL), *E. coli* TR (1 μg/mL), *E. coli* Trx (5 μM), NAD^+ (1 mM), NADPH (1 mM), or GSH (10 mM) as indicated. The reaction was started with 2 mM AcCoA or 2 mM *S*-acetylglutathione. When NEM, IAM, and MMTS were used, they were preincubated for 15 min at 37°C before the addition of AcCoA or *S*-acetylglutathione and then incubated for a further 3 or 6 hr at 37°C. The samples were mixed 1:1 with loading buffer containing 200 mM DTT before being run on a 12% SDS-PAGE gel. This DTT in the loading buffer reduces disulfides such as those generated by MMTS and remove cysteine-bound acetyl groups. Because the MMTS adduct is reduced by DTT in the loading buffer, the loss of signal does not result from an altered antigen-antibody interaction.

Small-Molecule ESI MS

20 μL of fresh HCO_3^- buffer (50 mM NH_4HCO_3 [pH 7.8]) were supplemented with mitochondrial membrane fragments (5 mg/mL), AcCoA (2 mM), GSH (10 mM), GSSG (5 mM), NEM (25 mM), IAM (25 mM), and MMTS (25 mM) as indicated. They were then incubated for up to 6 hr at 37°C before the addition of 980 μL of HCO_3^- buffer to quench the reaction. The samples were spun at 16,000 × *g* for 2 min, and the filtered supernatant was directly infused into a Xevo TQ-S triple quad mass spectrometer (Waters) at 50 μL/min. Samples were assessed using ESI in negative mode.

MALDI of Synthetic Peptides

Two synthetic peptides were designed, butyryl-RYAKGCASR-NH_2 (CysPep) and butyryl-RYAKGSASR-NH_2 (SerPep). The following features were designed into the synthetic peptides: one tyrosine for quantification by UV, one arginine at each end to facilitate MS of ion fragments, and a glycine between the lysine and the cysteine or serine for flexibility. The peptides also contained a butyrated N-terminal amide and a C-terminal amide, a common approach to improve stability and remove terminal charges that would not be present on most peptides within an intact protein. Four additional deuterated synthetic peptides were made as standards using deuterated butyric

acid: d7-butyryl-RYAKGCASR-NH_2 (d7-CysPep), d7-butyryl-RYAKGSASR-NH_2 (d7-SerPep), d7-butyryl-RYAK_{Ac}GCASR-NH_2 (d7-AcCysPep), and d7-butyryl-RYAK_{Ac}GSASR-NH_2 (d7-AcSerPep). For the reaction, 200 μM cysteine peptide, 200 μM serine peptide, 200 μM fresh AcCoA, and 1 mM fresh TCEP were coincubated together in 25 μL of fresh 10 mM NH_4HCO_3 (pH 7.8) at 37°C. After the incubation, a 10 μL aliquot of the reaction was added to 10 μL of either 40 mM DTT or H_2O. The DTT sample was incubated for a further 30 min at 37°C. To both aliquots, 475 μL of 0.1% trifluoroacetic acid (TFA) was added to quench further nucleophilic reactions, followed by 5 μL of a deuterated standard mix (50 μM d7-CysPep, 50 μM d7-SerPep, 50 μM d7-AcCysPep, and 50 μM d7-AcSerPep). For the 0 hr time point, AcCoA was added after 0.1% TFA.

The sample was spotted on the MALDI plate using the bottom-layer method. In total, 20 spectra with ten shots each were collected per spot, using a minimum intensity of 1,000 and a maximum intensity of 10,000 as the selection criteria. Peak intensities from three spots per experiment were quantified using mMass, and the concentration was calculated using summed intensities of the peptides and the d7 standards. Data are the average ± SEM of three experiments on separate days, each with three spots.

Statistics and Data Processing

Statistical significance was determined, usually relative to incubation with AcCoA, using a two-tailed Student's t test or one-way ANOVA followed by a Dunnett's multiple comparison test in Prism v.6. The overlap of the *S*-acylation (Gould et al., 2015) and *N*-acetylation (Rardin et al., 2013) datasets was determined using pgAdmin3. The peptides in the *N*-acetylation dataset contain two neighboring tryptic peptides joined together, because trypsin does not cleave at acetyllysine. Overlap occurred if either part of this miscleaved peptide was also in the *S*-acylation dataset.

See Supplemental Information for further details.

SUPPLEMENTAL INFORMATION

Supplemental Information includes Supplemental Experimental Procedures, three figures, and one table and can be found with this article online at http://dx.doi.org/10.1016/j.celrep.2017.02.018.

AUTHOR CONTRIBUTIONS

A.M.J. performed most of the experiments and data analysis and prepared the manuscript with M.P.M. M.P.M. was the grant holder. K.H. synthesized the peptides. A.R.H. supplied tissue. K.H. and I.M.F. gave suggestions about chemistry and MS, respectively. K.H., A.L., and S.D. processed samples for MS.

ACKNOWLEDGMENTS

We thank J. Zhu and J. Hirst for the bovine heart mitochondrial membranes and M. Harbour for generating MALDI-TOF-TOF spectra. This work was supported by the Medical Research Council (MC_U105663142) and by a Wellcome Trust investigator award (110159/Z/15/Z) to M.P.M.

Received: April 23, 2016
Revised: December 3, 2016
Accepted: February 3, 2017
Published: February 28, 2017

REFERENCES

Bizzozero, O.A., Bixler, H.A., and Pastuszyn, A. (2001). Structural determinants influencing the reaction of cysteine-containing peptides with palmitoyl-coenzyme A and other thioesters. Biochim. Biophys. Acta *1545*, 278–288.

Bracher, P.J., Snyder, P.W., Bohall, B.R., and Whitesides, G.M. (2011). The relative rates of thiol-thioester exchange and hydrolysis for alkyl and aryl thioalkanoates in water. Orig. Life Evol. Biosph. *41*, 399–412.

Cohen, T.J., Friedmann, D., Hwang, A.W., Marmorstein, R., and Lee, V.M. (2013). The microtubule-associated tau protein has intrinsic acetyltransferase activity. Nat. Struct. Mol. Biol. *20*, 756–762.

Davies, M.N., Kjalarsdottir, L., Thompson, J.W., Dubois, L.G., Stevens, R.D., Ilkayeva, O.R., Brosnan, M.J., Rolph, T.P., Grimsrud, P.A., and Muoio, D.M. (2016). The acetyl group buffering action of carnitine acetyltransferase offsets macronutrient-induced lysine acetylation of mitochondrial proteins. Cell Rep. *14*, 243–254.

Garland, P.B., Shepherd, D., and Yates, D.W. (1965). Steady-state concentrations of coenzyme A, acetyl-coenzyme A and long-chain fatty acyl-coenzyme A in rat-liver mitochondria oxidizing palmitate. Biochem. J. *97*, 587–594.

Gould, N.S., Evans, P., Martínez-Acedo, P., Marino, S.M., Gladyshev, V.N., Carroll, K.S., and Ischiropoulos, H. (2015). Site-specific proteomic mapping identifies selectively modified regulatory cysteine residues in functionally distinct protein networks. Chem. Biol. *22*, 965–975.

McDonnell, E., Peterson, B.S., Bomze, H.M., and Hirschey, M.D. (2015). SIRT3 regulates progression and development of diseases of aging. Trends Endocrinol. Metab. *26*, 486–492.

Pietrocola, F., Galluzzi, L., Bravo-San Pedro, J.M., Madeo, F., and Kroemer, G. (2015). Acetyl coenzyme A: a central metabolite and second messenger. Cell Metab. *21*, 805–821.

Rabbani, N., Xue, M., and Thornalley, P.J. (2014). Activity, regulation, copy number and function in the glyoxalase system. Biochem. Soc. Trans. *42*, 419–424.

Rardin, M.J., Newman, J.C., Held, J.M., Cusack, M.P., Sorensen, D.J., Li, B., Schilling, B., Mooney, S.D., Kahn, C.R., Verdin, E., and Gibson, B.W. (2013). Label-free quantitative proteomics of the lysine acetylome in mitochondria identifies substrates of SIRT3 in metabolic pathways. Proc. Natl. Acad. Sci. USA *110*, 6601–6606.

Requejo, R., Chouchani, E.T., James, A.M., Prime, T.A., Lilley, K.S., Fearnley, I.M., and Murphy, M.P. (2010). Quantification and identification of mitochondrial proteins containing vicinal dithiols. Arch. Biochem. Biophys. *504*, 228–235.

Roth, A.F., Wan, J., Bailey, A.O., Sun, B., Kuchar, J.A., Green, W.N., Phinney, B.S., Yates, J.R., 3rd, and Davis, N.G. (2006). Global analysis of protein palmitoylation in yeast. Cell *125*, 1003–1013.

Scott, I., Webster, B.R., Li, J.H., and Sack, M.N. (2012). Identification of a molecular component of the mitochondrial acetyltransferase programme: a novel role for GCN5L1. Biochem. J. *443*, 655–661.

Sharpley, M.S., Shannon, R.J., Draghi, F., and Hirst, J. (2006). Interactions between phospholipids and NADH:ubiquinone oxidoreductase (complex I) from bovine mitochondria. Biochemistry *45*, 241–248.

Talesa, V., Uotila, L., Koivusalo, M., Principato, G., Giovannini, E., and Rosi, G. (1989). Isolation of glyoxalase II from two different compartments of rat liver mitochondria. Kinetic and immunochemical characterization of the enzymes. Biochim. Biophys. Acta *993*, 7–11.

Thapa, P., Zhang, R.Y., Menon, V., and Bingham, J.P. (2014). Native chemical ligation: a boon to peptide chemistry. Molecules *19*, 14461–14483.

Vander Jagt, D.L. (1993). Glyoxalase II: molecular characteristics, kinetics and mechanism. Biochem. Soc. Trans. *21*, 522–527.

Wagner, G.R., and Payne, R.M. (2013). Widespread and enzyme-independent Nε-acetylation and Nε-succinylation of proteins in the chemical conditions of the mitochondrial matrix. J. Biol. Chem. *288*, 29036–29045.

Wagner, G.R., and Hirschey, M.D. (2014). Nonenzymatic protein acylation as a carbon stress regulated by sirtuin deacylases. Mol. Cell *54*, 5–16.

Weinert, B.T., Moustafa, T., Iesmantavicius, V., Zechner, R., and Choudhary, C. (2015). Analysis of acetylation stoichiometry suggests that SIRT3 repairs nonenzymatic acetylation lesions. EMBO J. *34*, 2620–2632.

Yan, Y., Harper, S., Speicher, D.W., and Marmorstein, R. (2002). The catalytic mechanism of the ESA1 histone acetyltransferase involves a self-acetylated intermediate. Nat. Struct. Biol. *9*, 862–869.

Cell Reports, Volume *18*

Supplemental Information

Non-enzymatic *N*-acetylation of Lysine Residues by AcetylCoA Often Occurs via a Proximal *S*-acetylated Thiol Intermediate Sensitive to Glyoxalase II

Andrew M. James, Kurt Hoogewijs, Angela Logan, Andrew R. Hall, Shujing Ding, Ian M. Fearnley, and Michael P. Murphy

Supplementary Materials

Non-enzymatic *N*-Acetylation of Lysine Residues by AcetylCoA Often Occurs via a Proximal *S*-Acetylated Thiol Intermediate Sensitive to Glyoxalase II

Andrew M. James[1,*], Kurt Hoogewijs[1,2,3], Angela Logan[1], Andrew R. Hall[1], Shujing Ding[1], Ian M. Fernley[1] and Michael P. Murphy[1,*,§]

[1]Medical Research Council Mitochondrial Biology Unit, Cambridge, CB2 0XY, UK

[2]Medical Research Council Laboratory of Molecular Biology, Cambridge, CB2 0QH, UK

[3]The Wellcome Trust Centre for Mitochondrial Research, Institute for Cell and Molecular Biosciences, The Medical School, Newcastle University, Newcastle upon Tyne, NE2 4HH, UK

[*]Correspondence: aj@mrc-mbu.cam.ac.uk; mpm@mrc-mbu.cam.ac.uk

SUPPLEMENTAL CALCULATIONS

Bovine heart mitochondrial membranes contain 53 nmol of exposed thiols/mg protein (Requejo et al., 2010). After 3h of incubation with 2 mM AcCoA, 1.75 nmol of acetyl was bound per mg of protein and of this 71% was sensitive to DTT and bound to cysteine (Figure 3A).

1.75 / 53 * 0.71 / 3 = 0.78% of exposed cysteines acetylated per h *in vitro* under these conditions

The frequency of lysine within protein (5.8%) is ~3-fold higher than cysteine (1.8%) (Trinquier and Sanejouand, 1998) and of these 4% of lysines and 47% of cysteines are buried (Schein, 1990). As we observed 29% of acetylation was DTT-insensitive (Figure 3A) it can be estimated that ~0.13% of exposed lysine residues are *N*-acetylated each hour.

0.78 / (5.8 / 1.8) * (0.96 / 0.53) * 0.29 = 0.13% exposed lysines acetylated per h *in vitro* under these conditions

Basal *N*-acetylation of the bovine heart mitochondrial membrane preparation is 17% of that observed after 6 h with 2 mM AcCoA (Figure 2F). This is equivalent to ~0.077% of exposed lysine residues.

0.13 * 6 * 0.17 = 0.13% exposed lysine amines basally acetylated *in vivo*

SUPPLEMENTAL EXPERIMENTAL PROCEEDURES

Materials – Recombinant human Glo2 (25 nmol/min/μg) and Sirt3 (2 pmol/min/μg) were from R&D Systems (Minneapolis, USA). ^{14}C-AcCoA was from American Radiolabelled Chemicals (St. Loius, USA). ^{14}H-GSH was from Perkin Elmer (Waltham, USA). ^{14}H-GSSG was prepared by mixing 500 nM ^{14}H-GSH with 25 mM GSSG for 1 h at 37˚C at pH 7.8. *S*-acetylglutathione (AcGS) was from Iris Biotech (Marktredwitz, Germany). Rabbit anti-acetyllysine (9441) was from Cell Signaling Technology (Danvers, USA). Rabbit anti-succinyllysine (PTM-401) was from PTM Biolabs (Chicago, USA). Mouse anti-NDUFB8 (ab110242) was from Abcam (Cambridge, UK). Anti-mouse and anti-rabbit fluorescent secondaries were from LI-COR Biosciences (Lincoln, USA). All other enzymes and chemicals were from Sigma. Bovine heart mitochondrial membrane fragments were prepared as described previously and stored at -80˚C until used (Sharpley et al., 2006). The bovine heart mitochondrial membranes were diluted 10-fold in appropriate buffer (see below), pelleted at 16,000 x g for 5 min and resuspended at 10 mg/mL in appropriate buffer to remove Tris. Solutions of TCEP, HA, GSH, GSSG, and AcGS were made up in buffer and neutralised prior to use.

Radioactive acetylation of protein - Bovine heart mitochondrial membrane fragments (5 mg/mL) were suspended in 20 μL of KP_i buffer (50 mM KH_2PO_4, 100 μM EDTA, 100 μM DTPA, pH 7.8) supplemented with *N*-ethyl maleimide (NEM; 25 mM), iodoacetamide (IAM; 25 mM), methylmethanethiosulphonate (MMTS; 25 mM), human Glo2 (1 μg/mL), human Sirt3 (10 μg/mL), 1 mM NAD^+ or 10 mM GSH as indicated. When NEM, IAM and MMTS were used they were preincubated with bovine heart mitochondrial membranes for 15 min at 37˚C prior to addition of AcCoA. The reaction was started with 50 μM ^{14}C-AcCoA and either 2 mM cold AcCoA or CoA and then they were incubated for up to 6h at either 0˚C or 37˚C. The reaction was quenched by the addition of 980 μL of KP_i buffer. To this 1 mL volume a large excess of dithiothreitol (DTT; 20 mM), hydroxylamine (HA; 50 mM), TCEP (20 mM) or GSH (20 mM) were sometimes added followed by a further 30 min incubation at 37˚C. Samples were spun at 16,000 x g for 10 min with the supernatant discarded and the pellet washed with a further 1 mL of 50 mM KP_i buffer. The supernatant was discarded and the inside of the tube dried with tissue. The pellet was resuspended in 50 μL of 20% (v/v) Triton X-100.

To control for unbound AcCoA in the pellet water space, 50 μM ^{14}C-AcCoA was omitted from the incubation and instead added immediately to the 1 mL of 50 mM KP_i buffer used to quench the reaction and before centrifugation. When ^{14}C-AcCoA was added after the incubation ^{14}C counts in the pellet water space were ~5% of the total at 6 h. EDTA and DTPA were omitted from the buffer for experiments with Glo2 (1 μg/mL) and Sirt3 (10 μg/mL). The specific activity of recombinant Sirt3 is much lower than recombinant Glo2, hence the addition of ten times as much protein.

Lysine acetylation - Bovine heart mitochondrial membrane fragments (5 mg/mL) were suspended in 20 µL of NaP_i buffer (50 mM NaH_2PO_4, 100 µM EDTA, 100 µM DTPA, pH 7.8) supplemented with NEM (25 mM), IAM (25 mM), MMTS (25 mM), human Glo2 (1 µg/mL), human Sirt3 (10 µg/mL), human Grx2 (1 µg/mL), yeast GR (0.1 µg/mL), *E.coli* TR (1 µg/mL), *E.coli* Trx (5 µM), 1 mM NAD^+, 1 mM NADPH or GSH (10 mM) as indicated. The reaction was started with 2 mM AcCoA or 2 mM *S*-acetyl-glutathione (AcGS). When NEM, IAM and MMTS were used they were preincubated for 15 min at 37˚C prior to addition of AcCoA or AcGS and then they were then incubated for 3 or 6h at 37˚C. The samples were mixed 1:1 with loading buffer containing 200 mM DTT before being run on a 12% SDS-PAGE gel. This DTT in the loading buffer will reduce disulphides such as those generated by MMTS and remove cysteine-bound acetyl groups. As the MMTS adduct is reduced by DTT in the loading buffer the loss of signal does not result from an altered antigen-antibody interaction. They were transferred to PVDF and blocked overnight at 4˚C with Odyssey® Blocking Buffer. The PVDF membrane was probed with 1/1000 rabbit anti-acetyllysine or rabbit anti-succinyllysine and 1/5000 mouse anti-NDUFB8 then visualised with anti-mouse and anti-rabbit fluorescent secondaries at 680 nm and 800 nm, respectively. Fluorescence intensity was measured using a LI-COR Odyssey® CLx near-infrared imaging system and Image Studio v4.0. EDTA and DTPA had no appreciable effect and were omitted for experiments with Glo2 (1 µg/mL) and Sirt3 (10 µg/mL).

Complex I-dependent NADH:hexaammineruthenium (HAR) activity - Bovine heart mitochondrial membrane fragments (5 mg/mL) were suspended in 20 µL of 50 mM NaH_2PO_4 (pH 7.8) supplemented with NEM (25 mM), IAM (25 mM), MMTS (25 mM). After incubation at 37˚C, 2 µL samples were taken and added to 18 µL of 20 mM Tris (pH 7.5) and snap frozen. After thawing, 5 µL of each diluted sample was added to per well of a 96-well plate. To this was added 200 µL of 200 µM NADH and 3.5 mM HAR in 20 mM Tris (pH 7.5) containing 4 mg/mL rotenone. The rate of NADH loss was measured at 340-380 nm using a Molecular Devices SpectraMax Plus 384 platereader. Unlike Complex I dependent NADH:Q activity, NADH:NAR activity is insensitive to alkylating reagents and rotenone, consequently loss of activity likely represents Complex I denaturation.

Small molecule ESI mass spectrometry - 20 µL of fresh bicarbonate buffer (50 mM NH_4HCO_3, pH 7.8) supplemented with bovine heart mitochondrial membranes (5 mg/mL), AcCoA (2 mM), GSH (10 mM), GSSG (5 mM), NEM (25 mM), IAM (25 mM), and MMTS (25 mM) as indicated. They were then incubated for up to 6h at 37˚C before the addition of 980 µL of bicarbonate buffer to quench the reaction. The samples were spun at 16,000 x g for 2 min and the filtered supernatant was directly infused into a Xevo TQ-S triple quad mass spectrometer (Waters) at 50 µL/min. Samples were assessed using electrospray ionisation in negative mode, with nitrogen as the curtain gas. Instrument parameters were: capillary voltage 3.1 kV; cone voltage 25 V; ion source temperature 150˚C. Data were acquired for one minute from 50 – 1200 m/z and total ion counts assessed using Masslynx software (Waters).

MALDI of synthetic peptides - Two synthetic peptides were designed, butyryl-RYAKGCASR-NH_2 (CysPep) and butyryl-RYAKGSASR-NH_2 (SerPep). The following features were designed into the synthetic peptides, one tyrosine for quantification by UV, one arginine at each end to facilitate MS of ion fragments, a glycine between the lysine and cysteine/serine for flexibility. The peptides also contained a butyrated N-terminal and a C-terminal amide, a common approach to improve stability and remove terminal charges that would not be present on most peptides within an intact protein. Peptides were synthesised manually on Rink amide ChemMatrix using Fmoc chemistry with 5 equivalents of Fmoc-amino acids or butyric acid, and PyBOP and DIPEA as coupling reagents. The obtained peptides were cleaved off the resin using TFA/DODT/TIS/H_2O (94:2.5:1:2.5), precipitated with diethyl ether and purified on HPLC (Waters XBridge Peptide BEH C18 OBD Prep Column, 300Å, 5 µm, 19 mm X 50 mm, 12 ml/min, from 100% water + 0.1% TFA to 100% Acetonitrile + 0.1% TFA). Fractions with purity >95% were collected and lyophilised. Four additional deuterated synthetic peptides were made as standards using deuterated butyric acid, d7-butyryl-RYAKGCASR-NH_2 (d7-CysPep), d7-butyryl-RYAKGSASR-NH_2 (d7-SerPep), d7-butyryl-RYAK_{Ac}GCASR-NH_2 (d7-AcCysPep) and d7-butyryl-RYAK_{Ac}GSASR-NH_2 (d7-AcSerPep). Trifluoroacetic (TFA) salts of the peptides were dissolved in water at ~1 mM then quantified by UV using tyrosine absorbance at 280 nm (1.49 $mM^{-1}.cm^{-1}$) and diluted to 500 µM in water. The peptides were stored like this at acidic pH to limit cysteine oxidation. For the reaction 200 µM cysteine peptide, 200 µM serine peptide, 200 µM fresh AcCoA and 1 mM fresh TCEP were coincubated in 25 µL fresh

bicarbonate buffer (10 mM NH_4HCO_3, pH 7.8) at 37˚C. After the incubation a 10 µL aliquot of the reaction was added to 10 µL of either 40 mM DTT or H_2O. The DTT sample was incubated for a further 30 min at 37˚C. To both aliquots 475 µL 0.1% TFA was added to quench further nucleophilic reactions followed by 5 µL of a deuterated standard mix (50 µM d7-cysteine peptide, 50 µM d7-serine peptide, 50 µM d7-acetylated cysteine peptide and 50 µM d7-acetylated serine peptide). For the 0 h time point, AcCoA was added after 0.1% TFA.

The sample was spotted on the MALDI-plate using the bottom-layer method. Matrix (0.75µL, 50% acetonitrile, 5 mg/ml α-cyano-4-hydroxycinnamic acid, 10 mM dibasic ammonium citrate, 0.1% TFA) was spotted on the plate and 0.75 µL sample was mixed in. The spot was left to dry at RT, after which another layer of 0.75 µL of matrix was added. In total 20 spectra with 10 shots each were collected per spot, using a minimum intensity of 1000 and a maximum of 10000 as selection criterion. Peak intensities from 3 spots per experiment were quantified using mMass and the concentration calculated using summed intensities of the peptides and the d7 standards. Data is the average ± SEM of 3 experiments on separate days each with 3 spots.

Statistics and data processing – Statistical significance was determined, usually relative to incubation with AcCoA, using a two-tailed Student's t-test or one-way ANOVA followed by a Dunnett's multiple comparison test in Prism v6. The overlap of the *S*-acylation (Gould et al., 2015) and *N*-acetylation (Rardin et al., 2013) datasets was determined using pgAdmin3. The peptides in the *N*-acetylation dataset contain two neighbouring tryptic peptides joined together as trypsin does not cleave at acetyllysine. Overlap occurred if either part of this miscleaved peptide was also in the *S*-acylation dataset.

SUPPLEMENTAL REFERENCES

Schein, C. H. 1990. Solubility as a function of protein structure and solvent components. *BioTechnology,* 8**,** 308-317.

Sharpley, M. S., Shannon, R. J., Draghi, F. & Hirst, J. 2006. Interactions between phospholipids and nadh:Ubiquinone oxidoreductase (complex i) from bovine mitochondria. *Biochemistry,* 45**,** 241-8.

Trinquier, G. & Sanejouand, Y. H. 1998. Which effective property of amino acids is best preserved by the genetic code? *Protein Eng,* 11**,** 153-69.

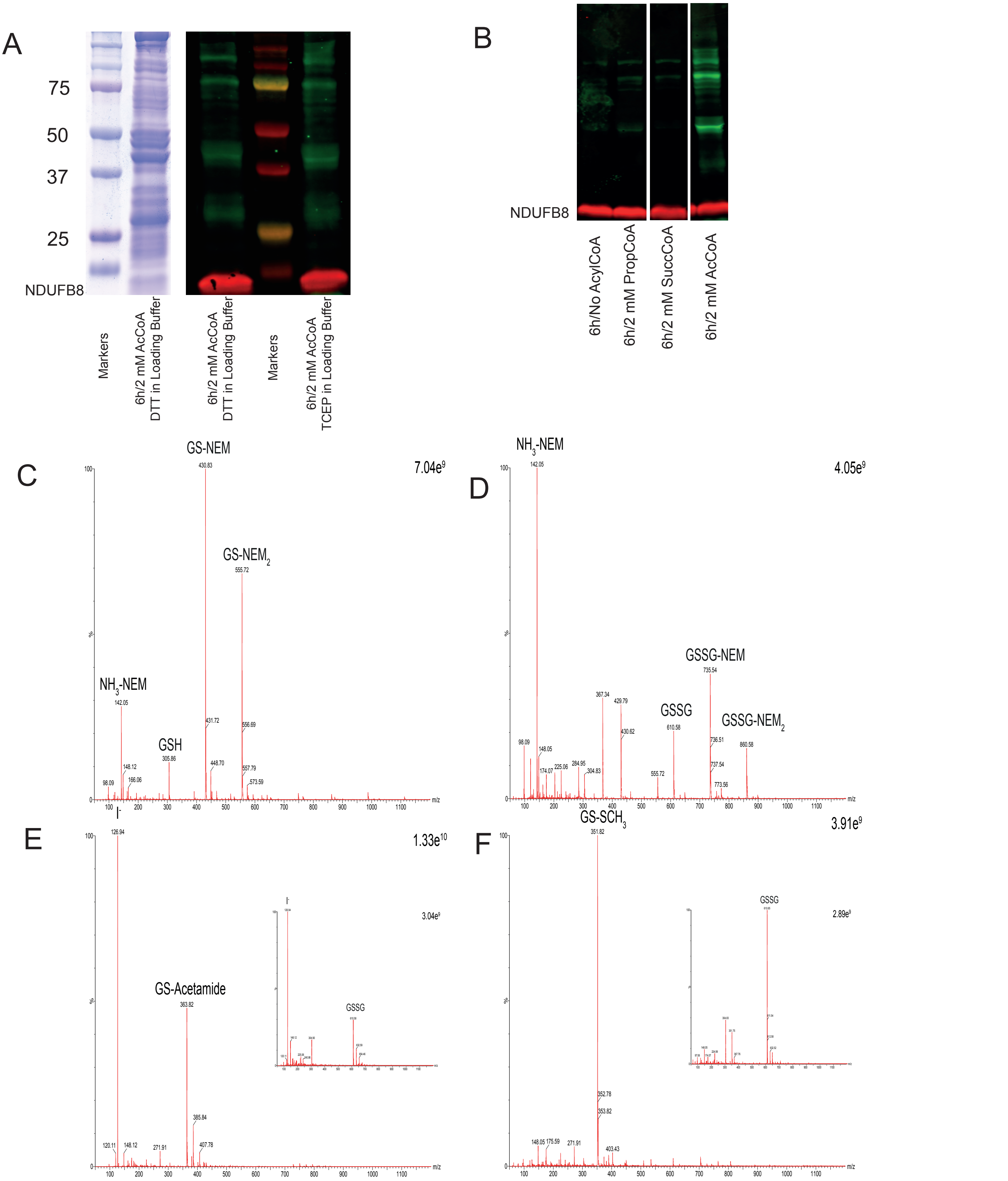

Figure S1. Specificity of antibodies and thiol reagents, Related to Figures 1 and 2.

A, the anti-acetyllysine antibody does not recognise *S*-acetylcysteine. Bovine heart mitochondrial membranes (5 mg/mL) were incubated with 2 mM AcCoA at 37˚C for 6 h. Samples were run on SDS-PAGE containing either 5 mM TCEP or 5 mM DTT. After SDS-PAGE total protein was visualised with coomassie or acetyllysine (green) and NDUFB8 (red) were visualised with fluorescent secondaries.
B, the anti-acetyllysine antibody does not recognise propionyllysine or succinyllysine. Bovine heart mitochondrial membranes (5 mg/mL) were incubated with 2 mM propionyl-CoA (PropCoA), succinyl-CoA (SuccCoA) or AcCoA at 37˚C for 6 h. Samples were run on SDS-PAGE with 100 mM DTT. After SDS-PAGE acetyllysine (green) and NDUFB8 (red) were visualised with fluorescent secondaries.
C-F, reactivity of NEM, IAM and MMTS with amines and thiols. GSH (10 mM) or GSSG (5 mM) were incubated in 50 mM NH_4HCO_3 (pH 7.8) buffer supplemented with 25 mM NEM, IAM or MMTS for 3 h at 37˚C. The sample was diluted 50-fold with NH_4HCO_3 (pH 7.8) buffer and GSH adducts were visualised by ESI MS in negative-ion mode. A and B, NEM also reacts with amines. NEM alkylates GSH and GSSG in two places, consistent with alkylation of one thiol and one amine on GSH or two amines on GSSG. It also alkylates buffer NH_3. C, IAM does not react with amines. IAM alkylates GSH in one place and does not alkylate GSSG (inset), consistent with alkylation of one thiol. D, MMTS does not react with amines. MMTS alkylates GSH in one place and does not alkylate GSSG (inset), consistent with alkylation of one thiol.

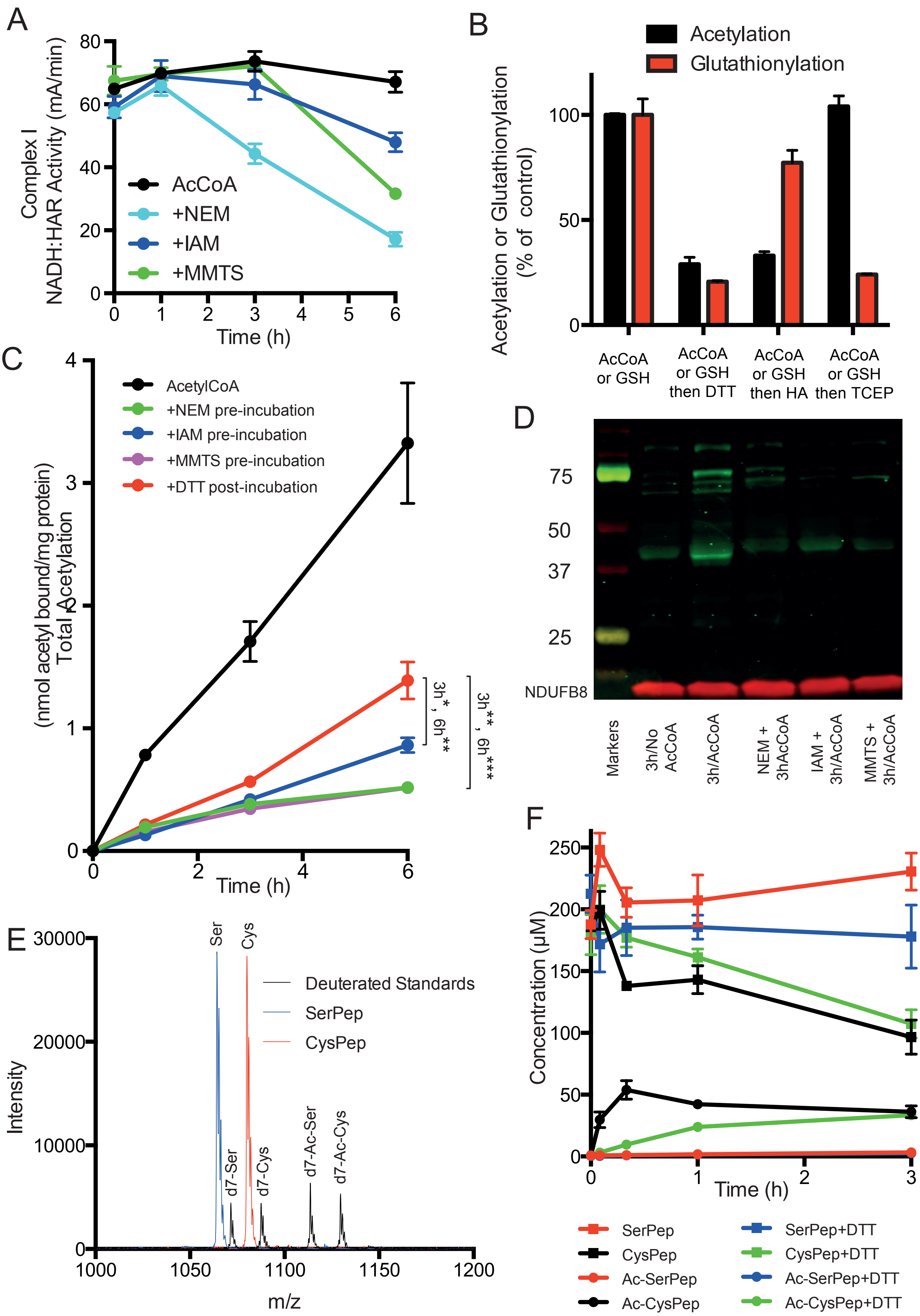

Figure S2. AcCoA acetylates lysine residues via a proximal acetylcysteine intermediate, Related to Figure 2.
A, sensitivity of complex I-dependent NADH:hexaammineruthenium (HAR) activity to thiol-blocking reagents. Bovine heart mitochondrial membrane fragments (5 mg/mL) were suspended in 20 μL of 50 mM NaH_2PO_4 (pH 7.8) supplemented with 25 mM NEM, IAM or MMTS. After incubation at 37˚C complex I-dependent NADH:HAR activity was measured. B, HA and TCEP selectively reduce thioesters and disulphides, respectively. Bovine heart mitochondrial membranes (5 mg/mL) were incubated with 2 mM ^{14}C-AcCoA or 5 mM ^{3}H-GSSG at 37˚C for up to 3 h. At the end of 3 h the reaction was quenched by addition of 1 mL of buffer containing 20 mM DTT, 20 mM TCEP or 50 mM HA. The sample was incubated for a further 30 min at 37˚C. The data are a percentage of the control mean ± SEM of at least 3 independent experiments. For ^{14}C-AcCoA the control mean was 1759 ± 12 nmol

^{14}C-Ac bound/mg protein. For ^{3}H-GSSG the control mean was 5174 ± 398 nmol ^{3}H-GS bound/mg protein. C, NEM, IAM and MMTS pre-incubation is significantly more effective at decreasing acetylation than post-incubation with a large excess of DTT. Bovine heart mitochondrial membranes (5 mg/mL) were incubated with 2 mM ^{14}C-AcCoA at 37˚C for up to 6 h. Some reactions were preincubated for 15 min at 37˚C with 25 mM NEM, IAM or MMTS. At the end of the incubation the reaction was quenched by addition of 1 mL of buffer. For DTT post-incubation this contained 20 mM DTT and the quenched sample was incubated for a further 30 min at 37˚C. The data are the mean ± SEM of at least 3 independent experiments. ****,** $p < 0.01$; *****,** $p < 0.001$. D, *N*-acetylation is sensitive to thiol-blocking reagents. Mitochondrial membranes were incubated with 2 mM AcCoA and either 25 mM NEM, IAM or MMTS at 37˚C for 3 h. After reducing SDS-PAGE acetyllysine (green) and NDUFB8 (red) were visualised. a proximal thiol facilitates lysine acetylation. E, MALDI of synthetic peptides. Two synthetic peptides were designed, butyryl-RYAKGCASR-NH_2 (red; CysPep) and butyryl-RYAKGSASR-NH_2 (blue; SerPep). Four additional deuterated synthetic peptides (black) were made as standards, d7-butyryl-RYAKGCASR-NH_2 (d7-CysPep), d7-butyryl-RYAKGSASR-NH_2 (d7-SerPep), d7-butyryl-RYAK_{Ac}GCASR-NH_2 (d7-AcCysPep) and d7-butyryl-RYAK_{Ac}GSASR-NH_2 (d7-AcSerPep). F, a cysteine residue facilitates acetylation of nearby lysine and serine residues. CysPep (200 μM) and SerPep (200 μM) were coincubated with 200 μM AcCoA. For some 20 mM DTT was added for 30 min after the reaction to differentiate acetylcysteine from acetyllysine or acetylserine. A mix of deuterated (d7) standards were then added to quantify acetylation (Ac-SerPep and Ac-CysPep) by MALDI-TOF. Acetylation was calculated relative to the acetyllysine containing d7-AcCysPep and d7-AcSerPep standards. The roughly equimolar loss of CysPep and gain of DTT-sensitive AcCysPep at 5 min suggests acetylcysteine containing AcCysPep can be quantified using the d7-AcCysPep standard.

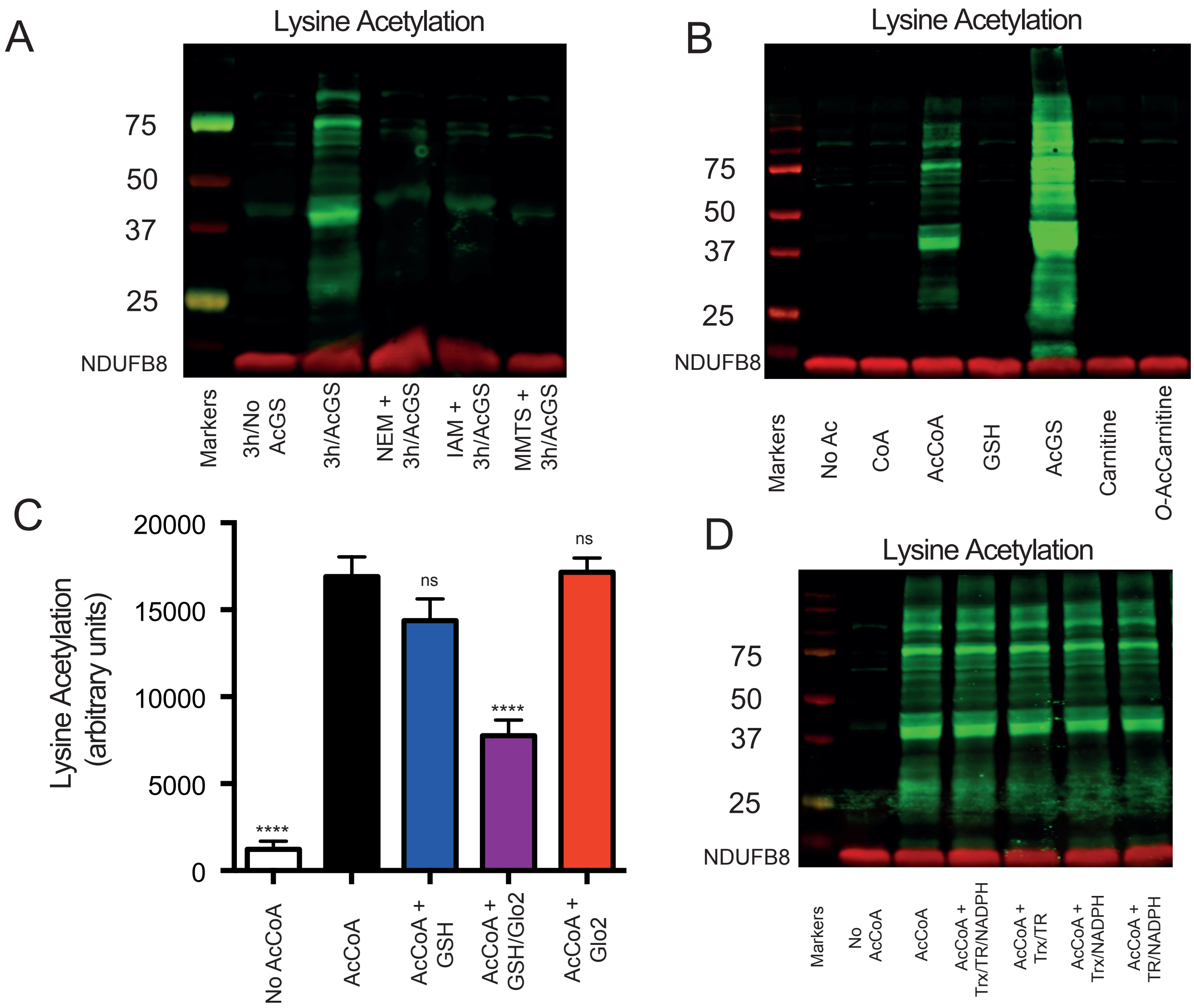

Figure S3. Acetylation is limited by glutathione and Glyoxalase II, Related to Figure 3.
A, *N*-acetylation is sensitive to thiol-blocking reagents. Mitochondrial membranes were incubated with 2 mM AcGS and either 25 mM NEM, IAM or MMTS at 37˚C for 3 h. After reducing SDS-PAGE acetyllysine (green) and NDUFB8 (red) were visualised. B, *O*-linked acetyl groups do not *N*-acetylate protein. Bovine heart mitochondrial membranes (5 mg/mL) were incubated with 2 mM CoA, AcCoA, GSH, AcGS, carnitine and *O*-acetylcarnitine at 37°C for 6 h. C, AcGS and AcCoA *N*-acetylate lysine residues and this is prevented by Glo2. Mitochondrial membranes were incubated with 2 mM AcCoA or 2 mM AcGS at 37˚C for 6 h. This was supplemented with GSH (10 mM) and/or Glo2 (1 µg/mL) during the 6h incubation as indicated. The data are the mean ± SEM of at least 3 independent experiments. ns, not significant; ****, $p < 0.0001$. D, thioredoxin reductase (TR) does not prevent *N*-acetylation of protein by AcCoA. Bovine heart mitochondrial membranes (5 mg/mL) were incubated with combinations of 2 mM AcCoA, 5 µM *E.coli* thioredoxin and 1 µg/mL *E.coli* TR, 1 mM NADPH for 6 h at 37˚C. After SDS-PAGE acetyllysine (green) and NDUFB8 (red) were visualised with fluorescent secondaries.

Table S1. Overlap of cysteine acylation and lysine acetylation, Related to Figure 4.
A list of mouse liver peptides containing both an acetyllysine (Rardin et al., 2013) and an acylcysteine (Gould et al., 2015). The acetyllysine dataset can be found in Table S4 (Rardin et al., 2013). The acylcysteine dataset can be found in Table S1 (Gould et al., 2015). The list includes peptides where the miscleaved acetyllysine containing peptide contains a cysteine residue that has a hydroxylamine-sensitive thioester modification. The sequences shown are longer than normal tryptic peptides because trypsin does not cleave at acetyllysine.
